# Vessel Density in the Macular and Peripapillary Areas in Preperimetric Glaucoma to Various Stages of Primary Open-Angle Glaucoma in Taiwan

**DOI:** 10.3390/jcm10235490

**Published:** 2021-11-23

**Authors:** Chung-Kuang Ko, Kuan-I Huang, Fang-Ying Su, Mei-Lan Ko

**Affiliations:** 1 Department of Neurology, China Medical University Hospital, Taichung 404, Taiwan; ko.chung.kuang@gmail.com; 2 Department of Ophthalmology, Shin-Kong Wu Ho-Su Memorial Hospital, Taipei 111, Taiwan; huangkuani@icloud.com; 3 Institute of Statistics, National Chiao Tung University, Hsinchu 300, Taiwan; fysu@hch.gov.tw; 4 Department of Ophthalmology, National Taiwan University Hospital, Hsinchu 300, Taiwan; 5 Biomedical Engineering and Environmental Sciences, National Tsing Hua University, Hsinchu 300, Taiwan

**Keywords:** glaucoma, macula, peripapillary, optical coherence tomography-angiography, vessel density, myopia

## Abstract

Peripapillary and macular vessel density (VD) are reduced in myopic non-glaucomatous eyes, the dynamic range of VD may be decreased by myopia, and whether VD measurement has the potential in differentiating stages of glaucoma severity in patients with myopic glaucoma remains questionable. This observational, cross-sectional study aimed to clarify the changes in peripapillary and macular VDs in preperimetric glaucoma (PPG) and primary open-angle glaucoma in the early, moderate, and late stages. A total of 1228 eyes from 661 participants (540 normal, 67 PPG, and 521 glaucomatous) were included. Participants underwent free blood tests at the internal medicine clinic to retrieve systemic data. Patients with glaucoma were grouped by disease severity, defined by glaucomatous visual field mean defect, including early-(224 eyes), moderate-(103 eyes), and late-stage glaucoma (194 eyes), and further divided into advanced (158 eyes) and terminal glaucoma (36 eyes). Macular VD, peripapillary VD, circumpapillary retinal nerve fiber layer (cpRNFL) thickness, and ganglion cell complex (GCC) thickness were evaluated and divided into superior and inferior parts. One-way analysis of variance was performed, followed by Tukey’s post-hoc test. The peripapillary VD was significantly different between the healthy and PPG groups and the early-, moderate-, and late-stage glaucoma subgroups (all *p* < 0.001). Peripapillary VD measurements are helpful in differentiating the various stages of glaucoma even in patients with myopic glaucoma.

## 1. Introduction

Glaucoma, which is the global leading cause of irreversible blindness in individuals aged 50 years and older, accounts for 11% of all blindness cases in 2020 [1]. In previous studies, Asians and Africans are disproportionately affected by glaucoma, and the number of patients with glaucoma worldwide is expected to increase to 111.8 million in 2040 [2]. Having a subtle chronic disease course, the pathogenesis of glaucoma has been theorized to correlate with microcirculatory defects in the retina. With regard to the prevention of glaucoma-related vision impairment, once detected, therapy for glaucoma can significantly preserve the visual field (VF) and slow its deterioration [3]. Early detection and treatment of glaucoma are very important in preserving vision, reducing morbidity, and diminishing the burden on healthcare systems. Optical coherence tomography angiography (OCTA) is a noninvasive, rapid, and high-resolution technique that provides three-dimensional images to illustrate blood supply status, different segmentations, and parameters to indicate the blood flow and microvasculature in the eyes. 

The mean defect (MD) in the VF is an important categorical severity classification system for glaucoma. OCTA parameters such as vessel density (VD) have been shown to be moderately or highly correlated with VF parameters [4]. For visual function decline in glaucomatous eyes, OCTA parameters are better biomarkers than OCT parameters since vascular loss has a stronger correlation with VF MD than with structural changes [5,6,7,8,9,10]. Some researchers suggest that OCTA may provide useful additional information for monitoring progression in later disease stages [4]. 

It has been reported that peripapillary and macular VDs are reduced in myopic non- glaucomatous eyes [11,12,13,14,15,16,17,18,19,20,21], and the simultaneous presence of myopia and open-angle glaucoma results in a greater level of microvascular attenuation than that with either pathology alone [22]. In myopic eyes, reduced VD may increase the susceptibility to vascular and age-related eye diseases [23,24]. The dynamic range of VD may be decreased by myopia, and whether VD measurement has the potential in differentiating stages of glaucoma severity in patients with myopic glaucoma remains questionable. 

The current study aimed to evaluate the changes in peripapillary and macular VDs in preperimetric glaucoma (PPG) and primary open-angle glaucoma (POAG) in various (early, moderate, advanced, and terminal) stages and to compare the potential of OCT and OCTA parameters in differentiating the stages of glaucoma severity, particularly in the myopic population. 

## 2. Materials and Methods

### 2.1. Study Participants

Patients aged 20 to 80 years who visited the ophthalmologic outpatient department of our hospital between June 2019 and February 2020 were included. This cross-sectional, observational study was conducted and approved by the institutional review board ((IRB) number: NTUHHCB 108-025-E) of the National Taiwan University Hospital Hsin-Chu Branch, Taiwan, and in accordance with the tenets of the Declaration of Helsinki (1964). Informed consent was obtained from all the participants in the study. 

All of the participants underwent a series of full ophthalmic examinations, including refractive error measurement by ARK-510A (Nidek Co., Gamagori, Japan), slit-lamp examination, gonioscopy, intraocular pressure (IOP) measurement by non-contact tonometer NT-530P (Nidek Co., Gamagori, Japan), open anterior chamber angle by gonioscopy, fundoscopy, visual field (VF) test by Humphrey Field Analyzer-840 (HFA-840; Carl Zeiss Meditec, Inc. Dublin, CA, USA), and axial length (AL) by AL-SCAN (Nidek Co., Gamagori, Japan). The bilateral eyes of every participant were evaluated and imaged. Then, they underwent a free blood test at the internal medicine clinic to retrieve systemic data, including mean arterial pressure (MAP), heart rate, serum triglyceride, high-density lipoprotein (HDL), low-density lipoprotein (LDL), serum sugar, HbA1C, alanine aminotransferase (ALT), creatinine, and uric acid. 

All controls had best-corrected visual acuity (BCVA) better than 12/20. To avoid poor image quality, the study excluded participants older than 60 years without previous cataract surgery. Further exclusion criteria were as follows: history of ocular surgery aside from uncomplicated cataract surgery; uveitis; ocular trauma; vitreoretinal diseases; non-glaucomatous optic neuropathy; and retinopathy, such as diabetic retinopathy, hypertensive retinopathy, maculopathy; any other known disease that may cause optic neuropathy, retinopathy or VF loss, unreliable VF (false-positive and false-negative rate > 15%, fixation losses > 20%), and unreliable OCTA image quality, such as a signal strength index less than 40. 

Control participants must show no evidence of retinal pathology or glaucoma in either eye, intraocular pressure of 21 mm Hg or less, no chronic ocular or systemic corticosteroid use, normal-appearing optic discs, and a normal result of VF examination. A normal VF was defined as a pattern standard deviation (PSD) within the 95% confidence limit and a normal glaucoma hemifield test result using a reliable VF test. 

In this study, preperimetric glaucoma (PPG) was defined on the basis of lesions in decreasing circumpapillary retinal nerve fiber layer (cpRNFL) thickness or ganglion cell complex (GCC) thickness by OCTA without glaucomatous VF abnormalities. Glaucomatous VF abnormalities were defined as follows: (1) PSD beyond 95% normal limits (*p* < 0.05) and glaucoma hemifield test beyond normal limits; (2) various glaucoma stages were defined with 24–2 models as early (−2.0 dB > MD ≥ −6 dB), moderate (−6 > MD ≥ −12 dB), and late stage further divided into advanced (−12 dB > MD ≥ −30 dB) and terminal stage (MD < −30 dB).

### 2.2. OCTA Measurements

All participants underwent OCTA (AngioVue, Optovue Inc., Fremont, CA, USA) examinations, which were reviewed manually to ensure correct segmentation and applicable quality. VD in the peripapillary area with a 4.5 × 4.5 mm^2^ scan size was divided into superior and inferior hemifields. VD in the macular area was centered on the fovea with a scan size of 3 × 3 mm^2^ with two concentric circles and diameters of 1 mm and 3 mm. It was further divided into center, superior, and inferior parts. The scan base was between the inner border of the internal limiting membrane and 10 μm above the outer border of the inner plexiform layer. The cpRNFL thickness was measured in an annulus centered on the optic disc with an outer and inner diameter of 4 mm and 2 mm, respectively. The GCC scan, including the nerve fiber, ganglion cell, and inner plexiform layers, covered a 7 mm × 7 mm area that is centered 1 mm temporal to the fovea. All images and data containing an optic nerve head analysis, including cup/disc ratio, rim area, and disc area, were automatically acquired using a built-in software.

### 2.3. Statistical Analysis

Categorical variables were expressed as percentages, while continuous variables as mean values and standard deviation (SD). Categorical variables were compared using the chi-square test and a two-sample independent *t*-test for other continuous variables. 

The propensity score (PS) was derived using logistic regression to model the probability of receipt in the control or glaucoma groups (PPG + glaucoma (G)) as a function of all the potential confounders listed in Table 1. Matching analysis based on the PS was used for different groups of glaucomatous and control participants to reduce selection biases between each pair of groups. Glaucoma groups (PPG + G) were matched to control groups (using the greedy matching algorithm) at a 1:1 ratio, 1:9 PS-matching for PPG groups and control groups, 1:1 PS-matching for glaucoma eye (excluding PPG) and control groups, and 1:5 PS-matching for glaucoma eye (excluding PPG) and PPG groups. Sex and age were used as covariates. 

The balance in baseline characteristics for different groups of glaucomatous and control participants among the PS-matched population was assessed using the Mantel– Haenszel test for sex and paired *t*-test for age.

To compare OCTA and OCT parameters within glaucoma severity groups, one-way analysis of variance was performed, followed by the Tukey’s post-hoc test. Two paired sample *t*-tests were used to compare the percentage loss of the superior and inferior regions of the macular and peripapillary VDs and cpRNFL and GCC thicknesses within the control, PPG (mean control − PPG)/mean control), and glaucoma groups (mean control − glaucoma)/mean control). 

Pearson’s correlation coefficient was used to evaluate the relationship between structural thickness and VD. Multivariable linear regression models using a generalized estimating equation (GEE) model were utilized for correlations between outcome and predictive variables, while adjusting for within-patient and inter-eye dependence. After adjusting for age, sex, and AL, the working correlation matrix was defined as exchangeable (compound symmetry); that is, the two eye measurements were assumed to be equally correlated and independent of the sequence. Statistical analysis was performed using the SAS (version 9.4; SAS Inc., Cary, NC, USA) and R (version 3.6.2) software. All tests were two-sided, and *p*-values < 0.05 were considered significant.

## 3. Results

**Figure 1:**
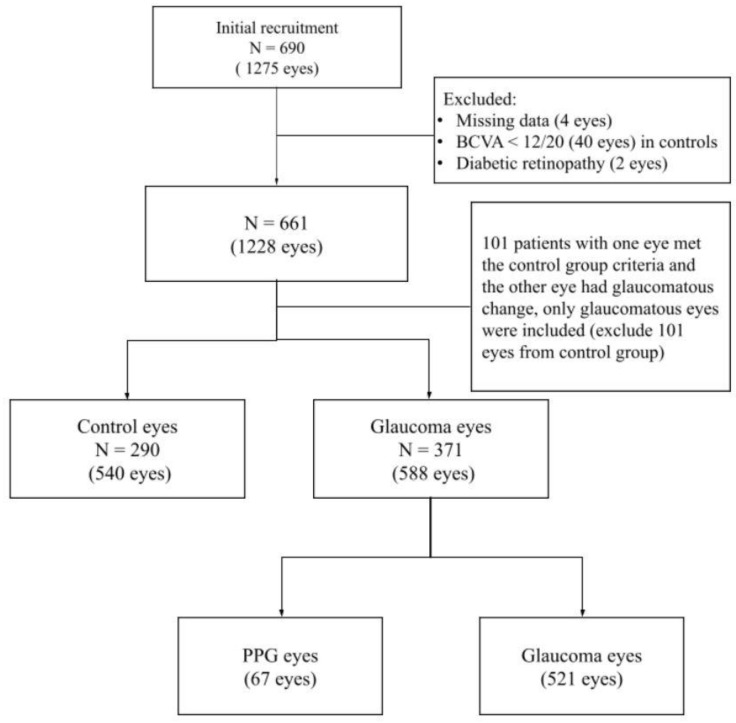
Flow chart of eligible eyes selection. After manual evaluation of the raw data to exclude ineligible eyes, we grouped the eyes into control eyes (540 eyes), PPG eyes (67 eyes), and glaucoma eyes (521 eyes) for the analysis. BCVA, best corrected visual acuity; PPG, pre-perimetric glaucoma.

Of the 1275 eyes of 690 participants aged 20–80 years that were initially enrolled in the study, 540 eyes from 290 control participants and 588 eyes from 371 glaucoma participants were eligible for the study. A flowchart for selecting the eligibility criteria is shown in Figure 1.

All raw data were manually evaluated, and four eyes with missing data, 40 eyes in controls with best-corrected visual acuity <0.6, and two eyes whose fundus photography showed diabetic retinopathy were excluded. Furthermore, 101 patients who had one eye that met the control group criteria, while the other eye with glaucomatous change, were excluded; hence, only glaucomatous eyes were included. A total of 1128 eyes were included in the database with SVD, cpRNFL thickness, and GCC thickness. For further multivariable linear regression models with GEE analysis, 58 eyes that were selected from those who only had a single eye that met the inclusion criteria were excluded. 

The demographics and characteristics of the control and glaucoma participants are summarized in Table 1 (Supplementary Table S1). No significant difference was found among the groups in terms of the prevalence of nephropathy, self-reported diabetes, self-reported hypertension, asthma, and CVA history (*p* > 0.05). The groups differed by age, sex, blood pressure, MAP, heart rate, HDL, Ante Cibum (AC) sugar, ALT, and eGFR (all *p* < 0.05). After matching for sex and age (Table 2, *N* = 200/200 in each group) (Supplementary Table S2), significant differences were not observed in the factors mentioned above in both groups.

Table 3 (Supplementary Table S2) summarizes the ophthalmic characteristics of the control and glaucoma groups after matching for sex and age. Significant differences were not found in eye laterality between the groups. The glaucoma group had significantly longer AL and thinner central corneal thickness (CCT) as expected, higher CD ratio, lower rim area, and worse VA, VF, VD, cpRNFL thickness, and GCC thickness (all *p* < 0.001) than those in the healthy group. A significant difference (*p* = 0.51) was not observed in the IOP between the two groups because of ethical and medical concerns. Moreover, the patients in the current study did not stop using antiglaucoma eye drops; therefore, patients with glaucoma in the present study should be more precisely described as those with medically treated glaucoma.

Table 4 and Table 5 (Supplementary Table S3) show the demographic and ophthalmic characteristics and ocular data, respectively, of the control, PPG, and glaucoma (G) groups after sex and age matching. The heart rate was significantly higher in the control group, whereas the AC sugar and HbA1C levels were significantly lower in the PPG group than those in the G group. The G group had significantly worse VA, thinner CCT, and longer AL than those in the control group. VF in the G group was significantly lower than that in the control and PPG groups. Significant differences in the hemifield macular VD, peripapillary VD, and cpRNFL and GCC thicknesses were noted among the three groups when compared in pairs (all *p* < 0.05) except for the center macular VD, which was only significantly lower in the G group than that in the control group.

Table 6 (Supplementary Table S4) summarizes the characteristics of the glaucomatous subgroups, which were divided according to the VF (MD) values, including the control (group 0), PPG (group 1), and early-(group 2), moderate-(group 3), and late-stage (group 4) glaucoma subgroups. Significant differences among the groups were not observed in terms of sex. Participants in the late-stage group were significantly older than those in the other groups, and their AL was significantly shorter. The peripapillary VD values were highest in the control eyes, followed by the PPG and early-, moderate-, and late-stage glaucoma eyes; all pairwise comparisons were statistically significant (*p* < 0.05, Tukey’s honestly significant difference). Superior and inferior macular VDs showed significant differences between the control, early, moderate, and late glaucoma groups but could not be differentiated between the PPG and early glaucoma groups.

The OCTA-based VD and OCT-based structural thickness of the two groups (advanced, terminal stage) are shown in Table 7 (Supplementary Table S5). No significant difference was noted between the two groups in terms of peripapillary and macular VDs, cpRNFL and GCC thickness, or CD ratio.

Multivariable linear regression models with the GEE for correlations between MD VF with OCTA parameters and OCT measurements are shown in Table 8. Positive correlations were found between MD VF with VD in the macula and peripapillary areas and cpRNFL and GCC thicknesses (all *p* < 0.001) except for the central macular VD. According to the *β*-value, the rates of increase were highest in macular VD (0.55%, 0.54% every dB), followed by peripapillary VD (0.47%, 0.53% every dB), GCC thickness (0.38 μm, 0.35 μm every dB), and cpRNFL thickness (0.24 μm, 0.25 μm every dB).

The superior and inferior hemifield asymmetries of the percentage loss of peripapillary and macular VDs and cpRNFL and GCC thickness were compared in the PPG and G groups (Table 9). In the PPG group, the GCC thickness showed significant intra-eye asymmetry, whereas the other factors did not. However, all factors were significantly more affected severely in the inferior hemifield of the glaucoma group (*p* < 0.001).

In Table 10, the r values of Pearson’s correlation coefficient between the cpRNFL and GCC thicknesses and VD were 0.636 to 0.674, and higher positive linear associations were noted in the peripapillary area than that in the macular area. The associations were slightly higher in the superior region than that in the inferior region.

In Table 11, positive linear correlations were found between superior and inferior GCC thicknesses with macular VD (all *p* < 0.001) and between superior and inferior cpRNFL thicknesses with peripapillary VD (*p* < 0.001). According to the β-value, the GCC and cpRNFL thicknesses of the superior macular, inferior macula, and superior peripapillary areas increased at a rate of 1.04 μm, 0.94 μm, 0.90 μm, and 0.91 μm for every percent of VD, respectively. The macular area has a higher β-value than that in the peripapillary area.

Although myopia is a risk factor for glaucoma, the glaucoma group showed a longer AL than the control group. However, we found that approximately half of the patients had more VF defects in eyes with higher myopia than in the inter-eye asymmetry of the same individual (Table 12).

## 4. Discussion

In the present investigation,

1. Significant differences in the hemifield macular VD and peripapillary VD were noted among the control, PPG, and glaucoma groups when compared in pairs (all *p* < 0.05) except for the center macular VD.
2. Peripapillary VD showed better potential in differentiating the various stages of glaucoma severity than macular VD even in the myopic population.
3. All parameters showed no significant differences between advanced and terminal glaucoma.
4. Positive correlations between VF defect with VD and thickness were noted, and rates of increase were highest in macular VD, followed by peripapillary VD, GCC thickness, and cpRNFL thickness.
5. The GCC thickness in the PPG group showed significant intra-eye asymmetry of percentage loss (*p* < 0.001), whereas peripapillary and macular VDs and cpRNFL thickness did not.
6. Only 50.6% of the patients had more VF defects in both eyes than that in the inter-eye asymmetry of the same individual.

Peripapillary VD measurement showed better potential in differentiating the stages (PPG, early, moderate, late) of glaucoma severity, whereas macular VD did not show a significant difference between the PPG and early-stage glaucoma groups in our study. However, the percent reduction in macular VD was significantly greater in the early glaucoma group than in the PPG group (all *p* < 0.05), as reported by Wang et al. [25]. The inconsistency may be attributed to our scan sizes being smaller than those of Wang’s study, and the most vulnerable macular area was found to be outside the central 3 × 3 mm^2^ area but inside the 6 × 6 mm^2^ area [26].

There was no significant difference between the parameters of advanced- (−30 dB < MD ≤ −12 dB) and terminal-stage (MD ≤ −30 dB) glaucoma. In the early stage of disease, both structural and functional data could help making judgements about the rate of progression. In the advanced stage, functional data, such as mean defect of VF, is more useful for follow-up in these cases. Severity in the advanced group (VF MD −20.2 ± 5.5 dB) may have reached the measurement floor of cpRNFL and GCC thickness, as supported by Moghimi et al. [27] and Phillips et al. [28]. Measurement floors of cpRNFL thickness and macular VD in our population may be higher than that in Americans [27]. Measuring the floor of peripapillary and macular VDs and cpRNFL and GCC thicknesses in the Asian population should be performed in the future. 

Mikelberg et al. found that 70% of glaucomatous eyes had initial damage limited to a single hemifield, and 57% still had a single hemifield defect at the completion of follow-up [29]. Therefore, the asymmetry of the percentage loss of VD and structural thickness in the PPG and G groups was compared. All parameters were significantly more affected severely in the inferior hemifield of the G group. In the PPG group, GCC thickness and hemifield asymmetry could be seen in the PPG stage but not in peripapillary and macular VDs, which was supported by previous studies. Chen et al. reported that macular ganglion cell–inner plexiform layer (GCIPL) thickness asymmetry measurements have diagnostic ability comparable to that of cpRNFL, GCIPL, and optic nerve head analysis for PPG [30]. Chang et al. also found that macular perfusion density asymmetry was not significantly different between the healthy and PPG groups [31]. 

The peripapillary and macular VDs and GCC and RNFL thicknesses were significantly more affected severely in the inferior hemifield than that in the superior hemifield of the glaucoma group (*p* < 0.001). The literature shows that the inferior region of the macula is particularly susceptible to glaucomatous damage [32]. Other studies have shown that VD superior/inferior asymmetry may permit a more reproducible, objective diagnosis in detecting early stages of glaucomatous changes [33,34]. As POAG progresses, the damage becomes multifocal and diffuse, and asymmetry becomes smaller in the severe stages of the disease [34]. Intra-eye VD asymmetry may have the potential to detect early glaucoma with less influence of interindividual variation in factors, such as age, sex, race, AL, hypertension, diabetes, and other systemic drug-related factors. In the future, it may be interesting to see if the VF hemifield test matches upper and lower VD, cpRNFL, and GCC differences in various stages of glaucoma.

A recent meta-analysis showed that individuals with myopia have a higher risk of developing open-angle glaucoma than those without myopia. The overall odds ratio (OR) was 1.95 for any myopia compared with emmetropia. The pooled ORs were 2.92 for moderate/high myopia and 1.59 for low myopia, with a cutoff value of −3 D [23]. In our study, the glaucoma group had longer AL than the control group, whereas in comparing the inter-eye asymmetry of the same individual, only 50.6% of the patients with glaucoma had more VF defects in eyes with higher myopia. In contrast, Lee et al. reported that in inter-eye comparisons, more myopic eyes in myopic normal-tension glaucoma demonstrate more severe VF [35]. However, the literature showed that the rates of VF progression in the myopic group (0.356 and 0.361 dB/y for 10° to 24°, respectively, and glaucoma hemifield test maps) were not significantly different from those in the non-myopic group (0.349 and –0.364 dB/y; *p* = 0.951 and 0.973, respectively) [36]. Other studies have reported that myopia may be a protective factor against optic disc/RNFL or VF progression in those with open-angle glaucoma [37,38,39]. Whether inter-eye asymmetrically higher myopia is associated with inter-eye asymmetrically progressive open-angle glaucoma remains questionable. Further studies are needed to clarify the association between the inter-eye asymmetry of myopia and open-angle glaucoma progression.

Our study population included more myopia patients and had a longer mean AL than those of other studies in healthy [27,40,41], PPG [40], and glaucoma groups [27,40]. The mean macular and peripapillary VD values of the glaucoma group in our study were lower than those in other studies [27,40]. However, for the healthy and PPG groups, no significant difference in the mean VD values was noted between our populations and other studies [27,40,41]. It has been reported that peripapillary and macular VD may be reduced in high myopia non-glaucoma patients [11,12,13,14,15,16,17,18,19,20,21]. While comparing the non-glaucoma population with that of Korean studies, our control group had shorter AL, lower macular and peripapillary VDs, and thinner RNFL [14,16]. Compared with studies conducted in China, our population had AL similar to that of moderate myopia defined in these studies, with higher macular VD in our group, lower peripapillary VD, and thinner GCC and peripapillary RNFL, which were lower and thinner than those of their high-myopia groups [15,17,20]. The dynamic range of VD was decreased by myopia, but using peripapillary VD to differentiate between the various stages of glaucoma severity is feasible. However, the simultaneous presence of myopia and open-angle glaucoma resulted in a greater level of microvascular attenuation than with either pathology alone [22]. The values of reduction related to glaucoma may have been overestimated. Considering the effect of myopia, VD may be more valuable in following glaucoma progression in each individual than comparing data between individuals in clinical practice, and different baseline values of parameters should not be neglected. 

According to the vascular theory of glaucoma, neural structure loss is a consequence of reduced ocular blood flow. Ischemia may damage the neural tissues [42,43]. On the contrary, the destruction of the neural tissue in glaucoma may lead to microvascular reduction through decreased metabolic demand [44]. The possible reasons why some studies failed to demonstrate this link might because of big variation of cross-sectional design. Longitudinal studies perhaps still cannot clarify whether neural tissue loss or vessel loss is the primary event since these events can be interdependent. 

There are some limitations to our study. First, our scan size was only 3 × 3 mm^2^, and a larger macular scan size (6 × 6 mm^2^) was reported to have a higher diagnostic accuracy [26,45]. A larger scan size of macular VD may help us understand more macular microvascular changes in the early stages of glaucoma. Second, the small sample size of the PPG group in this study may have induced selection bias in our population. Moreover, most systemic factors did not show differences between the control and glaucoma patients. Further studies with more participants may provide different results. Finally, since this was a cross-sectional study, the interindividual variability of measurements and disease progression could not be discussed. Longitudinal studies will help elucidate the pattern of microvascular damage in the various stages of glaucoma. 

## 5. Conclusions

In conclusion, the current study showed that peripapillary VD was significantly different between the healthy and PPG groups and the early-, moderate-, and late-stage glaucoma subgroups. Our results revealed that peripapillary VD measurement is helpful in differentiating the various stages of glaucoma severity, even in a myopic population.

## Figures and Tables

**Table 1:** Demographics and Characteristics of the Control and Glaucoma groups.

	Control (*N* = 290)	Glaucoma (*N* = 371)	*p*-Value
Age (years)	45.19 ± 14.23	51.95 ± 14.12	<0.001
<40	109 (37.6%)	76 (20.5%)	
40–60	142 (49.0%)	190 (51.2%)	
≥60	39(13.5%)	105 (28.3%)	
Gender			<0.001
Male	91 (31.4%)	231 (62.3%)	
Female	199 (68.6%)	140 (37.7%)	
SBP (mmHg)	123.81 ± 17.91	128.77 ± 18.61	<0.001
DBP (mmHg)	73.26 ± 12.35	76.07 ± 12.1	0.004
MAP (mmHg)	90.11 ± 13.31	93.63 ± 13.07	<0.001
HR (/min)	79.34 ± 12.46	75.31 ± 12.03	<0.0001
TG (mg/dL)	106.84 ± 70.33	117.08 ± 55.63	0.192
HDL (mg/dL)	56.04 ± 13.75	51.86 ± 15.29	0.024
LDL (mg/dL)	113.89 ± 33.05	108.98 ± 30.45	0.292
AC sugar (mg/dL)	94.86 ± 14.73	104.39 ± 23.19	<0.001
HbA1c (%)	5.98 ± 0.65	6.01 ± 0.88	0.798
ALT (U/L)	21.54 ± 18.05	27.21 ± 19.37	0.007
Creatinine (mg/dL)	0.9 ± 1.22	0.95 ± 0.77	0.641
eGFR (mL/min/1.73 m^2^)	99.83 ± 22.24	91.92 ± 21.34	0.001
Uric Acid(mg/dL)	5.24 ± 1.51	5.94 ± 1.36	<0.001

Note. SBP, systolic blood pressure; DBP, diastolic blood pressure; MAP, mean arterial pressure; HR, heart rate; TG, triglyceride; HDL, high-density lipoprotein (cholesterol); LDL, low-density lipoprotein (cholesterol); ALT, aspartate aminotransferase; eGFR, estimated glomerular filtration rate.

**Table 2:** Demographics and Characteristics of the Control and Glaucoma groups after Gender and Age Matching.

	Control (*N* = 200)	Glaucoma (*N* = 200)	*p*-Value
Age (years)	47.4 ± 15.2	49.3 ± 14.7	0.08
<40	62 (31.0%)	51 (25.5%)	
40–60	101 (50.5%)	107 (53.5%)	
≥60	37 (18.5%)	42 (21.0%)	
Gender			0.19
Male	88 (44.0%)	75 (37.5%)	
Female	112 (56.0%)	125 (62.5%)	
SBP (mmHg)	124.7 ± 17.9	126.9 ± 19.4	0.23
DBP (mmHg)	74.2 ± 11.8	73.3 ± 11.5	0.45
MAP (mmHg)	91.0 ± 12.9	91.2 ± 13.1	0.91
HR (/min)	78.4 ± 13.1	76.3 ± 12.4	0.10
TG (mg/dL)	114.7 ± 84.5	102.9 ± 52.2	0.29
HDL (mg/dL)	56.1 ± 15.0	54.7 ± 15.2	0.56
LDL (mg/dL)	107.2 ± 31.0	104.9 ± 31.2	0.70
AC sugar (mg/dL)	96.5 ± 16.1	101.9 ± 24.2	0.10
HbA1c (%)	6.0 ± 0.7	5.8 ± 0.7	0.25
ALT (U/L)	23.6 ± 20.9	25.8 ± 21.1	0.47
Creatinine (mg/dL)	1.0 ± 1.3	0.9 ± 0.4	0.45
eGFR (mL/min/1.73 m^2^)	97.1 ± 21.4	95.6 ± 22.4	0.63
Uric Acid(mg/dL)	5.5 ± 1.8	5.6 ± 1.5	0.93

Note. SBP, systolic blood pressure; DBP, diastolic blood pressure; MAP, mean arterial pressure; HR, heart rate; TG, triglyceride; HDL, high-density lipoprotein (cholesterol); LDL, low-density lipoprotein (cholesterol); ALT, aspartate aminotransferase; eGFR, estimated glomerular filtration rate.

**Table 3:** Ocular Data of the Control and Glaucoma groups after Gender and Age Matching.

	Control Eyes (*N* = 343)	Glaucoma Eyes (*N* = 343)	*p*-Value
OD/OS			0.76
OD	173 (50.4%)	177 (51.6%)	
OS	170 (49.6%)	166 (48.4%)	
VA (logMAR)	0.1 ± 0.2	0.3 ± 0.6	<0.001
AL (mm)	25.1 ± 1.7	25.7 ± 2.1	<0.001
<24	100 (29.2%)	75 (22.0%)	<0.001
24–25.9	145 (42.3%)	101 (29.6%)	
≥26	98 (28.6%)	165 (48.4%)	
IOP (mmHg)	14.9 ± 3.6	14.7 ± 4.1	0.509
CCT (µm)	547.1 ± 33.2	535.4 ± 37.9	<0.001
VF: Mean Defect	−1.2 ± 1.8	−8.2 ± 8.3	<0.001
Macular VD (%)			
Superior	50.1 ± 4.7	43.8 ± 7.4	<0.001
Center	18.7 ± 6.4	16.4 ± 7.0	<0.001
Inferior	49.6 ± 4.7	41.4 ± 8.1	<0.001
Peripapillary VD (%)			
Superior	51.6 ± 5.0	40.5 ± 10.7	<0.001
Inferior	52.7 ± 5.0	37.9 ± 10.5	<0.001
cpRNFL Thickness (µm)			
Superior	100.0 ± 9.6	78.2 ± 15.5	<0.001
Inferior	96.1 ± 9.1	72.0 ± 15.1	<0.001
GCC Thickness (µm)			
Superior	95.6 ± 5.5	78.4 ± 12.5	<0.001
Inferior	95.0 ± 5.5	72.3 ± 13.2	<0.001
Cup/Disc Ratio (%)	51.2 ± 18.6	79.2 ± 16.0	<0.001
Rim Area (0.01 mm^2^)	131.1 ± 36.9	75.9 ± 39.8	<0.001
Disc Area (0.01 mm^2^)	203.5 ± 49.4	212.1 ± 61.5	0.046

Note. OD, right eye; OS, left eye; VA, visual acuity; AL, axial length; CCT, central corneal thickness; VF, visual field; VD, vessel density; cpRNFL, circumpapillary retinal nerve fiber layer; GCC, ganglion cell complex.

**Table 4:** Demographics and Clinical Characteristics after Gender and Age Matching for Groups of Glaucomatous Subjects and Control Subjects.

	A vs. B *N* = 288:32		A vs. C *N* = 314:314		B vs. C *N* = 62:310		*p*-Value		
							a	b	c
Age	44.7 ± 13.9	48.6 ± 15.7	47.7 ± 14.4	48.2 ± 12.9	46.9 ± 12.2	50.6 ± 14.1	0.12	0.52	0.33
<40	112 (38.9%)	9 (28.1%)	81 (25.8%)	95 (30.2%)	15 (24.2%)	75 (24.2%)	0.24	0.95	0.20
40–60	142 (49.3%)	18 (56.3%)	177 (56.4%)	150 (47.8%)	40 (64.5%)	163 (52.6%)			
≥60	34 (11.8%)	5 (15.6%)	56 (17.8%)	69 (22.0%)	7 (11.3%)	72 (23.2%)			
Gender							1.00	1.00	0.23
Male	72 (25.0%)	8 (25.0%)	156 (49.7%)	156 (49.7%)	43 (69.3%)	190 (61.3%)			
Female	216 (75.0%)	24 (75.0%)	158 (50.3%)	158 (50.3%)	19 (30.7%)	120 (38.7%)			
SBP	122.5 ± 17.7	130.3 ± 18.4	126.2 ± 18.4	125.9 ± 17.5	129.5 ± 16.0	129.0 ± 18.6	0.03	0.85	0.82
DBP	72.0 ± 12.0	76.8 ± 13.0	75.4 ± 12.0	74.5 ± 11.7	78.6 ± 12.5	76.0 ± 12.3	0.03	0.35	0.13
MAP	88.9 ± 13.0	94.7 ± 13.5	92.4 ± 13.2	91.7 ± 12.5	95.6 ± 12.7	93.6 ± 13.1	0.02	0.51	0.28
HR	79.4 ± 12.4	75.5 ± 9.8	78.8 ± 12.3	74.7 ± 11.5	74.9 ± 10.1	75.5 ± 12.3	0.08	<0.001	0.71
TG	105.7 ± 69.9	102.3 ± 45.6	122.7 ± 87.1	113.1 ± 49.7	108.6 ± 45.0	116.9 ± 57.2	0.64	0.27	0.52
HDL	55.9 ± 13.5	55.2 ± 12.4	52.4 ± 14.1	52.5 ± 14.8	52.0 ± 11.7	50.4 ± 15.5	0.77	0.96	0.63
LDL	113.7 ± 32.8	115.2 ± 35.1	113.6 ± 34.1	109.1 ± 29.1	119.2 ± 27.4	113.4 ± 31.7	0.89	0.35	0.47
AC sugar	94.1 ± 14.3	93.4 ± 8.1	96.9 ± 14.1	100.3 ± 19.8	95.4 ± 8.1	104.2 ± 22.5	0.70	0.12	0.001
HbA1c	6.0 ± 0.7	5.1 ± 0.3	6.0 ± 0.5	6.1 ± 1.1	5.4 ± 0.6	6.1 ± 0.9	0.02	0.33	0.01
ALT	20.2 ± 17.1	32.0 ± 21.9	22.2 ± 11.9	23.8 ± 16.2	31.1 ± 21.7	25.7 ± 18.3	0.03	0.38	0.18
Creatinine	0.9 ± 1.2	0.8 ± 0.2	1.0 ± 1.5	0.9 ± 0.8	0.9 ± 0.2	0.9 ± 0.8	0.49	0.47	0.27
eGFR	100.9 ± 21.3	99.2 ± 16.6	95.0 ± 21.4	95.9 ± 21.2	97.9 ± 17.7	93.0 ± 21.2	0.37	0.71	0.26
Uric Acid	5.1 ± 1.3	5.3 ± 1.3	5.7 ± 1.6	5.9 ± 1.3	5.9 ± 1.3	6.0 ± 1.4	0.51	0.46	0.85

Group A, control eyes; Group B, pre-perimetric glaucoma eyes; Group C, glaucoma eyes; a *p*-value, A vs. B; b *p*-value, A vs. C; c *p*-value, B vs. C; Unit. Age (years); SBP (mmHg); DBP (mmHg); MAP (mmHg); HR (/min); TG (mg/dL); HDL (mg/dL); LDL (mg/dL); AC sugar (mg/dL); HbA1c (%); ALT (U/L); Creatinine (mg/dL); eGFR (mL/min/1.73 m^2^); Uric Acid (mg/dL); Abbreviations. SBP, systolic blood pressure; DBP, diastolic blood pressure; MAP, mean arterial pressure; HR, heart rate; TG, triglyceride; HDL, high-density lipoprotein (cholesterol); LDL, low-density lipoprotein (cholesterol); ALT, aspartate aminotransferase; eGFR, estimated glomerular filtration rate.

**Table 5:** Ocular Data after Gender and Age Matching for Groups of Glaucomatous subjects and Control subjects.

	A vs. B *N* = 288:32		A vs. C *N* = 314:314		B vs. C *N* = 62:310		*p*-Value		
							a	b	c
OD/OS							0.68	0.58	0.68
OD	151 (52.4%)	18 (56.3%)	160 (51.0%)	153 (48.7%)	30 (48.4%)	159 (51.3%)			
OS	137 (47.6%)	14 (43.7%)	154 (49.0%)	161 (51.3%)	32 (51.6%)	151 (48.7%)			
VA (logMAR)	0.1 ± 0.2	0.2 ± 0.6	0.1 ± 0.2	0.3 ± 0.5	0.3 ± 0.6	0.4 ± 0.7	0.37	<0.001	0.20
AL (mm)	25.2 ± 1.8	25.6 ± 1.8	25.1 ± 1.8	25.7 ± 2.1	25.9 ± 1.9	25.6 ± 2.4	0.22	<0.001	0.37
<24	78 (27.1%)	7 (21.9%)	97 (30.9%)	71 (22.8%)	13 (21.0%)	86 (27.8%)			
24–25.9	121 (42.0%)	8 (25.0%)	118 (37.6%)	100 (32.0%)	16 (25.8%)	87 (28.2%)			
26	89 (30.9%)	17 (53.1%)	99 (31.5%)	141 (45.2%)	33 (53.2%)	136 (44.0%)			
IOP (mmHg)	14.6 ± 3.4	14.9 ± 3.3	14.8 ± 3.6	14.8 ± 4.0	15.1 ± 4.0	14.7 ± 3.8	0.66	0.84	0.45
CCT (µm)	543.9 ± 35.9	542.2 ± 31.0	544.0 ± 35.4	536.4 ± 34.1	530.7 ± 44.7	536.5 ± 36.0	0.80	0.007	0.34
VF	−1.4 ± 1.8	−1.4 ± 1.5	−1.2 ± 1.9	−9.0 ± 8.6	−1.0 ± 1.3	−10.3 ± 9.0	0.98	<0.001	<0.001
Macular VD (%)									
Superior	50.1 ± 4.8	47.3 ± 5.1	49.9 ± 5.3	43.5 ± 7.1	46.4 ± 6.7	42.9 ± 7.5	0.002	<0.001	<0.001
Center	18.4 ± 6.8	18.0 ± 6.4	18.6 ± 6.3	16.1 ± 7.0	17.8 ± 6.2	16.0 ± 6.9	0.72	<0.001	0.06
Inferior	49.5 ± 5.1	46.1 ± 4.8	49.4 ± 5.4	41.0 ± 7.7	45.7 ± 6.1	40.2 ± 8.0	0.001	<0.001	<0.001
Peripapillary VD (%)									
Superior	51.7 ± 5.2	47.1 ± 6.4	51.3 ± 5.3	39.1 ± 10.6	47.3 ± 5.9	37.9 ± 10.6	<0.001	<0.001	<0.001
Inferior	52.7 ± 5.4	48.5 ± 5.2	52.4 ± 5.4	36.6 ± 10.2	47.4 ± 5.1	35.4 ± 10.3	<0.001	<0.001	<0.001
cpRNFL Thickness (µm)									
Superior	101.0 ± 10.1	85.6 ± 10.2	100.8 ± 10.0	76.0 ± 16.0	86.6 ± 10.3	75.1 ± 15.4	<0.001	<0.001	<0.001
Inferior	96.9 ± 9.4	85.1 ± 9.8	96.3 ± 8.9	69.3 ± 15.0	81.5 ± 10.7	67.8 ± 13.4	<0.001	<0.001	<0.001
GCC Thickness (µm)									
Superior	96.0 ± 5.9	86.3 ± 7.4	95.8 ± 5.4	76.6 ± 12.2	85.9 ± 8.0	76.3 ± 13.0	<0.001	<0.001	<0.001
Inferior	95.4 ± 5.9	83.5 ± 7.3	95.1 ± 5.6	70.5 ± 12.5	81.3 ± 8.1	70.0 ± 12.8	<0.001	<0.001	<0.001
C/D Ratio	49.8 ± 19.5	66.5 ± 18.6	51.5 ± 19.4	80.7 ± 15.6	70.5 ± 14.8	82.5 ± 13.4	<0.001	<0.001	<0.001
Rim Area	133.3 ± 37.2	109.3 ± 63.7	130.8 ± 37.1	72.0 ± 39.1	98.1 ± 50.8	69.8 ± 37.9	0.04	<0.001	<0.001
Disc Area	203.5 ± 48.4	218.9 ± 56.2	204.2 ± 49.4	212.6 ± 60.1	212.8 ± 48.8	216.4 ± 59.5	0.09	0.06	0.66

Group A, control eyes; Group B, pre-perimetric glaucoma eyes; Group C, glaucoma eyes; a *p*-value, A vs. B; b *p*-value, A vs. C; c *p*-value, B vs. C; Unit. VA (logMAR); VF (mean deviation); VD (%); thickness (µm); C/D ratio (%); Rim area (0.01 mm^2^); Disc area (0.01 mm^2^); Abbreviation. OD, right eye; OS, left eye; VA, visual acuity; AL, axial length; CCT, central corneal thickness; VF, visual field; VD, vessel density; cpRNFL, circumpapillary retinal nerve fiber layer; GCC, ganglion cell complex; C/D Ratio, cup to disc ratio.

**Table 6:** One Way Analysis of OCTA parameters and OCT measurement among the glaucoma severity groups.

	Group 0 *N* = 540	Group 1 *N* = 67	Group 2 *N* = 224	Group 3 *N* = 103	Group 4 *N* = 194	*p*-Value	Tukey Test
Age (years)	44.4 ± 13.8	45.7 ± 12.7	48.3 ± 12.2	51.3 ± 12.8	56.1 ± 14.5	<0.001	4 > 0, 1, 2, 3
Axial Length (mm)	25.2 ± 1.7	25.9 ± 1.8	25.9 ± 1.8	26.3 ± 2.8	25.1 ± 2.2	<0.001	1, 2, 3 > 0, 4
Gender						0.463	
Male	169 (31.3%)	43 (64.2%)	137 (61.2%)	69 (67.7%)	132 (68.0%)		
Female	371 (68.7%)	24 (35.8%)	87 (38.8%)	33 (32.4%)	62 (32.0%)		
Macular VD (%)							
Superior	50.3 ± 4.8	46.6 ± 6.5	46.1 ± 6.0	41.2 ± 6.6	38.1 ± 7.5	<0.001	0 > 1, 2 > 3 > 4
Center	18.4 ± 6.4	18.1 ± 6.1	17.0 ± 6.6	14.7 ± 6.1	14.5 ± 7.0	<0.001	0 > 2; 0, 1 ,2 > 3, 4
Inferior	49.7 ± 5.0	46.0 ± 5.9	43.7 ± 6.8	38.0 ± 7.2	35.2 ± 7.7	<0.001	0 > 1, 2 > 3 > 4
Peripapillary VD (%)							
Superior	51.8 ± 4.9	47.4 ± 5.8	43.6 ± 8.4	37.1 ± 10.2	29.5 ± 9.2	<0.001	0 > 1 > 2 > 3 > 4
Inferior	52.9 ± 5.2	47.6 ± 5.0	41.5 ± 8.1	33.2 ± 9.2	26.9 ± 7.8	<0.001	0 > 1 > 2 > 3 > 4
cpRNFL Thickness (µm)							
Superior	100.8 ± 9.7	86.8 ± 10.4	81.2 ± 13.5	74.0 ± 14.6	67.1 ± 14.9	<0.001	0 > 1 > 2 > 3 > 4
Inferior	96.8 ± 9.0	82.0 ± 10.7	74.6 ± 12.8	65.2 ± 14.0	61.1 ± 13.4	<0.001	0 > 1 > 2 > 3 > 4
GCC Thickness (µm)							
Superior	95.9 ± 5.8	86.0 ± 7.8	81.1 ± 10.2	73.9 ± 12.6	69.4 ± 12.4	<0.001	0 > 1 > 2 > 3 > 4
Inferior	95.3 ± 5.7	81.5 ± 8.0	75.0 ± 12.1	65.9 ± 10.4	64.2 ± 11.1	<0.001	0 > 1 > 2 > 3 > 4
Cup/Disc Ratio (%)	49.7 ± 19.5	69.5 ± 16.0	77.5 ± 14.0	83.4 ± 13.7	89.5 ± 11.0	<0.001	0 < 1 < 2 < 3 < 4
Rim Area (0.01 mm^2^)	133.3 ± 36.7	100.1 ± 50.2	78.5 ± 33.9	69.8 ± 38.2	51.6 ± 30.3	<0.001	0 > 1 > 2, 3 > 4
Disc Area (0.01 mm^2^)	203.4 ± 48.6	213.9 ± 49.8	209.5 ± 51.8	221.0 ± 81.9	216.7 ± 60.6	0.006	

Group 0, control eyes; Group 1, pre-perimetric glaucoma eyes; Group 2, early glaucoma eyes with −2 dB > VF(MD) ≥ −6 dB; Group 3, moderate glaucoma eyes with −6 dB > VF(MD) ≥ −12 dB; Group 4, late glaucoma eyes with VF(MD) < −12 dB; OD, right eye; OS, left eye; VA, visual acuity; AL, axial length; CCT, central corneal thickness; VF, visual field; VD, vessel density; cpRNFL, circumpapillary retinal nerve fiber layer; GCC, ganglion cell complex; MD, mean deviation.The OCTA-based VD and OCT-based structural thickness of the two groups (advanced, terminal stage) are shown in Table 7. No significant difference was noted between the two groups in terms of peripapillary and macular VDs, cpRNFL and GCC thickness, or CD ratio.

**Table 7:** Comparison of advanced or terminal glaucoma with OCTA parameters and OCT measurement.

	Advanced Glaucoma (*N* = 158)	Terminal Glaucoma (*N* = 36)	*p*-Value
Age (years)	56.06 ± 14.44	56.47 ± 14.91	0.879
Male subjects	110(69.62)	22(61.11)	0.323
Macular VD (%)			
Superior	38.13 ± 7.44	37.84 ± 7.93	0.845
Center	13.98 ± 6.48	17.19 ± 8.88	0.064
Inferior	35.15 ± 7.56	35.64 ± 8.36	0.759
Peripapillary VD (%)			
Superior	29.68 ± 8.84	28.70 ± 11.02	0.635
Inferior	26.86 ± 7.33	27.16 ± 9.92	0.876
cpRNFL Thickness (µm)			
Superior	66.99 ± 13.61	67.75 ± 19.56	0.827
Inferior	60.94 ± 13.20	61.72 ± 14.21	0.753
GCC Thickness (µm)			
Superior	69.58 ± 11.95	68.56 ± 14.44	0.667
Inferior	63.96 ± 10.89	65.26 ± 11.81	0.536
Cup/Disc Ratio (%)	90.04 ± 9.79	87.11 ± 15.17	0.274
Rim Area (0.01 mm^2^)	49.92 ± 25.06	59.03 ± 46.49	0.262
Disc Area (0.01 mm^2^)	217.80 ± 59.97	211.69 ± 63.65	0.587
AL (mm)	25.07 ± 2.19	24.98 ± 2.24	0.818
VF (mean defect)	−20.24 ± 5.49	−31.71 ± 0.87	<0.001
VA (logMAR)	0.55 ± 0.79	0.91 ± 1.20	0.148

Note. Advanced glaucoma, −12 dB > VF(MD) ≥ −30 dB; Terminal glaucoma, VF(MD) < −30 dB; Abbreviation. VD, vessel density; cpRNFL, circumpapillary retinal nerve fiber layer; GCC, ganglion cell complex; VA, visual acuity; AL, axial length.

**Table 8:** Multivariable linear regression models with generalized estimating equation (GEE) model * for correlations between visual field in mean defect with OCTA parameters and OCT measurement.

	β	SE	*p*-Value
Macular VD (%)			
Superior	0.55	0.05	<0.001
Center	0.16	0.10	0.123
Inferior	0.54	0.05	<0.001
Peripapillary VD (%)			
Superior	0.47	0.04	<0.001
Inferior	0.53	0.03	<0.001
cpRNFL Thickness (µm)			
Superior	0.24	0.03	<0.001
Inferior	0.25	0.03	<0.001
GCC Thickness (µm)			
Superior	0.38	0.03	<0.001
Inferior	0.35	0.03	<0.001
Cup/Disc Ratio (%)	−0.23	0.04	<0.001
Rim Area (0.01 mm^2^)	0.07	0.01	<0.001
Disc Area (0.01 mm^2^)	0.00	0.01	0.936

OCTA, optical coherence tomography angiography; VD, vessel density; cpRNFL, circumpapillary retinal nerve fiber layer; GCC, ganglion cell complex. * Adjusted for age, gender, axial length.

**Table 9:** Comparison of percentage loss hemifield asymmetry of corresponding regions of OCTA parameters between pre-perimetric glaucoma eyes and glaucoma eyes.

	PPG ^†^ *N* = 67	*p*-Value *	Glaucoma ^‡^ *N* = 521	*p*-Value *
Macular VD				
Superior	0.07 ± 0.13	0.94	0.16 ± 0.15	<0.001
Inferior	0.08 ± 0.12		0.20 ± 0.16	
Peripapillary VD				
Superior	0.09 ± 0.11	0.34	0.28 ± 0.21	<0.001
Inferior	0.10 ± 0.10		0.35 ± 0.20	
cpRNFL Thickness				
Superior	0.14 ± 0.10	0.36	0.26 ± 0.15	<0.001
Inferior	0.15 ± 0.11		0.30 ± 0.15	
GCC Thickness				
Superior	0.10 ± 0.08	<0.001	0.21 ± 0.13	<0.001
Inferior	0.14 ± 0.08		0.27 ± 0.13	

PPG, pre-perimetric glaucoma; VD, vessel density; cpRNFL, circumpapillary retinal nerve fiber layer; GCC, ganglion cell complex; * Two sample pair *t*-test; ^†^ PPG group = (Mean Control –PPG)/Mean Control; ^‡^ G group = (Mean Control – G)/Mean Control.

**Table 10:** Pearson correlation coefficient between OCTA parameters with OCT measurement in same region.

		**r**	***p*-Value**
Superior	GCC Thickness Macular VD	0.658	<0.001
Inferior	GCC Thickness Macular VD	0.636	<0.001
Superior	cpRNFL Thickness Peripapillary VD	0.674	<0.001
Inferior	cpRNFL Thickness Peripapillary VD	0.666	<0.001

OCTA, optical coherence tomography angiography; VD, vessel density; cpRNFL, circumpapillary retinal nerve fiber layer; GCC, ganglion cell complex.

**Table 11:** Multivariable linear regression models with generalized estimating equation (GEE) model * for correlations between OCTA parameters with OCT measurement in same region: change in structural thickness related with VD.

		β	SE	*p*-Value
Superior	GCC Thickness Macular VD	1.04	0.05	<0.001
Inferior	GCC Thickness Macular VD	0.94	0.05	<0.001
Superior	cpRNFL Thickness Peripapillary VD	0.90	0.06	<0.001
Inferior	cpRNFL Thickness Peripapillary VD	0.91	0.05	<0.001

OCTA, optical coherence tomography angiography; VD, vessel density; cpRNFL, circumpapillary retinal nerve fiber layer; GCC, ganglion cell complex; * Adjusted for age, sex, axial length.

**Table 12:** Proportion of higher myopia with more VF defect in all patients with glaucoma.

Glaucoma Subjects, *N* = 318	*N*	%
Higher myopia with more VF defect ^a^	161	50.6
Others ^b^	157	49.4

VF, visual field; ^a^ In bilateral eyes: AL differences (mm) > 0 and VF differences (dB) < 0 or AL differences (mm) < 0 and VF differences (dB) > 0; ^b^ In bilateral eyes: AL differences (mm) ≤ 0 and VF differences (dB) ≤ 0 or AL differences (mm) ≥ 0 and VF differences (dB) ≥ 0.

## Data Availability

The data presented in this study are available in supplementary materials.

## Supplementary Materials

The following are available online at https://www.mdpi.com/article/10.3390/jcm10235490/s1, Table S1 : Demographics and Characteristics of the Control and Glaucoma groups, Table S2 : Demographics, Characteristics and Ocular Data of the Control and Glaucoma groups after Gender and Age Matching, Table S3 : Demographics, Clinical Characteristics and Ocular Data after Gender and Age Matching for Groups of Glaucomatous Subjects and Control Subjects, Table S4 : One Way Analysis of OCTA parameters and OCT measurement among the glaucoma severity groups, Table S5 : Comparison of advanced or terminal glaucoma with OCTA parameters and OCT measurement.

## References

[1] GBD 2019 Blindness and Vision Impairment Collaborators, Vision Loss Expert Group of the Global Burden of Disease Study (2021). Causes of blindness and vision impairment in 2020 and trends over 30 years, and prevalence of avoidable blindness in relation to VISION 2020: The Right to Sight: An analysis for the Global Burden of Disease Study. Lancet Glob. Health.

[2] Tham Y.-C., Li X., Wong T.Y., Quigley H.A., Aung T., Cheng C. (2014). Global prevalence of glaucoma and projections of glaucoma burden through 2040: A systematic review and meta-analysis. Ophthalmology.

[3] Garway-Heath D.F., Crabb D.P., Bunce C., Lascaratos G., Amalfitano F., Anand N., Azuara-Blanco A., Bourne R.R., Broadway D.C., A Cunliffe I. (2015). Latanoprost for open-angle glaucoma (UKGTS): A randomised, multicentre, placebo-controlled trial. Lancet.

[4] Werner A.C., Shen L.Q. (2019). A Review of OCT Angiography in Glaucoma. Semin. Ophthalmol..

[5] Van Melkebeke L., Barbosa-Breda J., Huygens M., Stalmans I. (2018). Optical Coherence Tomography Angiography in Glaucoma: A Review. Ophthalmic Res..

[6] Yarmohammadi A., Zangwill L.M., Diniz-Filho A., Saunders L.J., Suh M.H., Wu Z., Manalastas P.I.C., Akagi T., Medeiros F.A., Weinreb R.N. (2017). Peripapillary and Macular Vessel Density in Patients with Glaucoma and Single-Hemifield Visual Field Defect. Ophthalmology.

[7] Jia Y., Wei E., Wang X., Zhang X., Morrison J.C., Parikh M., Lombardi L.H., Gattey D.M., Armour R.L., Edmunds B. (2014). Optical Coherence Tomography Angiography of Optic Disc Perfusion in Glaucoma. Ophthalmology.

[8] Shin J.W., Lee J., Kwon J., Choi J., Kook M.S. (2017). Regional vascular density–visual field sensitivity relationship in glaucoma according to disease severity. Br. J. Ophthalmol..

[9] Yarmohammadi A., Zangwill L.M., Diniz-Filho A., Suh M.H., Yousefi S., Saunders L.J., Belghith A., Manalastas P.I.C., Medeiros F.A., Weinreb R.N. (2016). Relationship between Optical Coherence Tomography Angiography Vessel Density and Severity of Visual Field Loss in Glaucoma. Ophthalmology.

[10] Chen H.S.-L., Liu C.-H., Wu W.-C., Tseng H.-J., Lee Y.-S. (2017). Optical Coherence Tomography Angiography of the Superficial Microvasculature in the Macular and Peripapillary Areas in Glaucomatous and Healthy Eyes. Investig. Opthalmol. Vis. Sci..

[11] Ucak T., Icel E., Yilmaz H., Karakurt Y., Tasli G., Ugurlu A., Bozkurt E. (2020). Alterations in optical coherence tomography angiography findings in patients with high myopia. Eye (Lond.).

[12] Yang Y., Wang J., Jiang H., Yang X., Feng L., Hu L., Wang L., Lü F., Shen M. (2016). Retinal Microvasculature Alteration in High Myopia. Investig. Opthalmol. Vis. Sci..

[13] Milani P., Montesano G., Rossetti L., Bergamini F., Pece A. (2018). Vessel density, retinal thickness, and choriocapillaris vascular flow in myopic eyes on OCT angiography. Graefe’s Arch. Clin. Exp. Ophthalmol..

[14] Min C.H., Al-Qattan H.M., Lee J.Y., Kim J.-G., Yoon Y.H., Kim Y.J. (2020). Macular Microvasculature in High Myopia without Pathologic Changes: An Optical Coherence Tomography Angiography Study. Korean J. Ophthalmol..

[15] Li Y., Miara H., Ouyang P., Jiang B. (2018). The Comparison of Regional RNFL and Fundus Vasculature by OCTA in Chinese Myopia Population. J. Ophthalmol..

[16] Sung M.S., Lee T.H., Heo H., Park S.W. (2017). Clinical features of superficial and deep peripapillary microvascular density in healthy myopic eyes. PLoS ONE.

[17] He J., Chen Q., Yin Y., Zhou H., Fan Y., Zhu J., Zou H., Xu X. (2019). Association between retinal microvasculature and optic disc alterations in high myopia. Eye (Lond.).

[18] Cheng D., Chen Q., Wu Y., Yu X., Shen M., Zhuang X., Tian Z., Yang Y., Wang J., Lu F. (2019). Deep perifoveal vessel density as an indicator of capillary loss in high myopia. Eye (Lond.).

[19] Hassan M., Sadiq M.A., Halim M.S., Afridi R., Soliman M.K., Sarwar S., Agarwal A., Do D.V., Nguyen Q.D., Sepah Y.J. (2017). Evaluation of macular and peripapillary vessel flow density in eyes with no known pathology using op-tical coherence tomography angiography. Int. J. Retin. Vitr..

[20] Wu Q., Chen Q., Lin B., Huang S., Wang Y., Zhang L., Lin H., Wang J., Lu F., Shen M. (2020). Relationships among retinal/choroidal thickness, retinal microvascular network and visual field in high my-opia. Acta Ophthalmol..

[21] Al-Sheikh M., Phasukkijwatana N., Dolz-Marco R., Rahimi M., Iafe N.A., Freund K.B., Sadda S.R., Sarraf D. (2017). Quantitative OCT Angiography of the Retinal Microvasculature and the Choriocapillaris in Myopic Eyes. Investig. Opthalmol. Vis. Sci..

[22] Suwan Y., Fard M.A., Geyman L.S., Tantraworasin A., Chui T.Y., Rosen R.B., Ritch R. (2018). Association of Myopia With Peripapillary Perfused Capillary Density in Patients With Glaucoma: An Op-tical Coherence Tomography Angiography Study. JAMA Ophthalmol..

[23] Haarman A.E.G., Enthoven C.A., Tideman J.W.L., Tedja M.S., Verhoeven V.J.M., Klaver C.C.W. (2020). The Complications of Myopia: A Review and Meta-Analysis. Investig. Opthalmol. Vis. Sci..

[24] Benavente-Pérez A., Hosking S.L., Logan N.S., Broadway D.C. (2010). Ocular blood flow measurements in healthy human myopic eyes. Graefe’s Arch. Clin. Exp. Ophthalmol..

[25] Wang Y., Xin C., Li M., Swain D.L., Cao K., Wang H., Wang N. (2020). Macular vessel density versus ganglion cell complex thickness for detection of early primary open-angle glaucoma. BMC Ophthalmol..

[26] Hood D.C. (2017). Improving our understanding, and detection, of glaucomatous damage: An approach based upon optical co-herence tomography (OCT). Prog. Retin. Eye Res..

[27] Moghimi S., Bowd C., Zangwill L.M., Penteado R.C., Hasenstab K., Hou H., Ghahari E., Manalastas P.I.C., Proudfoot J., Weinreb R.N. (2019). Measurement Floors and Dynamic Ranges of OCT and OCT Angiography in Glaucoma. Ophthalmology.

[28] Phillips M.J., Dinh-Dang D., Bolo K., Burkemper B., Lee J.C., LeTran V.H., Chang B.R., Grisafe D.J., Chu Z., Zhou X. (2021). Steps to Measurement Floor of an Optical Microangiography Device in Glaucoma. Am. J. Ophthalmol..

[29] Mikelberg F.S., Drance S.M. (1984). The Mode of Progression of Visual Field Defects in Glaucoma. Am. J. Ophthalmol..

[30] Chen M.-J., Yang H.-Y., Chang Y.-F., Hsu C.-C., Ko Y.-C., Liu C.J.-L. (2019). Diagnostic ability of macular ganglion cell asymmetry in Preperimetric Glaucoma. BMC Ophthalmol..

[31] Chang P.-Y., Wang J.-Y., Wang J.-K., Yeh S.-C., Chang S.-W. (2020). Asymmetry analysis of optical coherence tomography angiography macular perfusion density measurements in preperimetric and perimetric glaucoma. Sci. Rep..

[32] Hood D.C., Raza A., de Moraes C.G.V., Liebmann J.M., Ritch R. (2013). Glaucomatous damage of the macula. Prog. Retin. Eye Res..

[33] Shakrawal J., Sihota R., Azad S., Kamble N., Dada T. (2021). Circumpapillary optical coherence tomography angiography differences in perimetrically affected and unaffected hemispheres in primary open-angle glaucoma and the preperimetric fellow eye. Indian J. Ophthalmol..

[34] Hong K.L., Burkemper B., Urrea A.L., Chang B.R., Lee J.C., LeTran V.H., Chu Z., Zhou X., Xu B.Y., Wong B.J. (2021). Hemiretinal Asymmetry in Peripapillary Vessel Density in Healthy, Glaucoma Suspect, and Glaucoma Eyes. Am. J. Ophthalmol..

[35] Lee E.J., Han J.C., Kee C. (2017). Intereye comparison of ocular factors in normal tension glaucoma with asymmetric visual field loss in Korean population. PLoS ONE.

[36] Lee J.R., Kim S., Lee J.Y., Back S., Lee K.S., Kook M.S. (2016). Is Myopic Optic Disc Appearance a Risk Factor for Rapid Progression in Medically Treated Glaucomatous Eyes With Confirmed Visual Field Progression?. J. Glaucoma.

[37] Qiu C., Qian S., Sun X., Zhou C., Meng F. (2015). Axial Myopia Is Associated with Visual Field Prognosis of Primary Open-Angle Glaucoma. PLoS ONE.

[38] Lee J.Y., Sung K.R., Han S., Na J.H. (2015). Effect of Myopia on the Progression of Primary Open-Angle Glaucoma. Investig. Opthalmology Vis. Sci..

[39] Naito T., Yoshikawa K., Mizoue S., Nanno M., Kimura T., Suzumura H., Umeda Y., Shiraga F. (2016). Relationship between visual field progression and baseline refraction in primary open-angle glaucoma. Clin. Ophthalmol..

[40] Hou H., Moghimi S., Zangwill L.M., Shoji T., Ghahari E., Penteado R.C., Akagi T., Manalastas P.I.C., Weinreb R.N. (2019). Macula Vessel Density and Thickness in Early Primary Open-Angle Glaucoma. Am. J. Ophthalmol..

[41] You Q.S., Chan J.C.H., Ng A.L.K., Choy B.K.N., Shih K.C., Cheung J.J.C., Wong J.K.W., Shum J.W.H., Ni M., Lai J.S.M. (2019). Macular Vessel Density Measured with Optical Coherence Tomography Angiography and Its Associations in a Large Population-Based Study. Investig. Opthalmol. Vis. Sci..

[42] Flammer J., Orgül S., Costa V.P., Orzalesi N., Krieglstein G.K., Serra L.M., Renard J.-P., Stefánsson E. (2002). The impact of ocular blood flow in glaucoma. Prog. Retin. Eye Res..

[43] Mansouri K. (2016). Optical coherence tomography angiography and glaucoma: Searching for the missing link. Expert Rev. Med. Devices.

[44] Akil H., Chopra V., Al-Sheikh M., Falavarjani K.G., Huang A.S., Sadda S.R., A Francis B. (2017). Swept-source OCT angiography imaging of the macular capillary network in glaucoma. Br. J. Ophthalmol..

[45] Wan K., Lam A.K.N., Leung C.K.-S. (2018). Optical Coherence Tomography Angiography Compared With Optical Coherence Tomography Macular Measurements for Detection of Glaucoma. JAMA Ophthalmol..

