# Supplementary material for: Vessel Density in the Macular and Peripapillary Areas in Preperimetric Glaucoma to Various Stages of Primary Open-Angle Glaucoma in Taiwan
Source: J Clin Med. 2021 Nov 23;10(23):5490. doi: 10.3390/jcm10235490 (PMC8658219; doi:10.3390/jcm10235490)
Supplement: Supplementary file 1 [file jcm-10-05490-s001.zip › Supplementary Table S1.pdf]

Demographics and Characteristics of the Control and Glaucoma groups

	control group patients(N=290)								galucoma group patients(N= 371)									
	N	(%)	Means	SD	min	max	95%CI		N	(%)	Means	SD	min	max	95%CI		P-value	
Age	290		45.19	14.23	20	81	43.55	46.83	371		51.95	14.12	20	80	50.5	53.39	<0.0001	
<40	109	37.6							76	20.5							<0.0001	Chisq
40-60	142	49.0							190	51.2								
>=60	39	13.5							105	28.3								
Sex																	<0.0001	Chisq
M	91	31.4							231	62.3								
F	199	68.6							140	37.7								
SBP	289		123.81	17.91	72	185	121.73	125.88	369		128.77	18.61	90	205	126.86	130.67	0.0006	
DBP	289		73.26	12.35	44	113	71.83	74.69	369		76.07	12.1	44	113	74.83	77.31	0.0035	
MAP	289		90.11	13.31	57.33	134	88.57	91.65	369		93.63	13.07	60.33	140.33	92.3	94.97	0.0007	
HR	289		79.34	12.46	49	121	77.89	80.78	369		75.31	12.03	45	117	74.08	76.54	<0.0001	
Kidney disease																	0.4668	Chisq
N	284	97.9							359	97.0								
Y	6	2.1							11	3.0								
H/T																	0.3788	Chisq
N	245	84.5							303	81.9								
Y	45	15.5							67	18.1								
DM																	0.0598	Chisq
N	274	94.5							335	90.5								
Y	16	5.5							35	9.5								
Bad sleep																	0.1753	Chisq
N	273	94.1							338	91.4								
Y	17	5.9							32	8.7								
asthma																	0.5271	Chisq
N	283	97.6							358	96.8								
Y	7	2.4							12	3.2								
CVA																	0.6893	Chisq
N	273	94.1							350	94.9								
Y	17	5.9							19	5.2								
TG	141		106.84	70.33	31	549	95.13	118.55	118		117.08	55.63	25	316	106.94	127.23	0.1918	
HDL	135		56.04	13.75	30	93	53.7	58.38	113		51.86	15.29	30	103	49.01	54.71	0.0242	
LDL	90		113.89	33.05	36	208	106.97	120.81	97		108.98	30.45	33	193	102.84	115.12	0.2918	
AC sugar	150		94.86	14.73	68	177	92.48	97.24	135		104.39	23.19	80	207	100.44	108.33	<0.0001	
HbA1C	41		5.98	0.65	5	8.1	5.77	6.18	89		6.01	0.88	4.5	10.5	5.83	6.2	0.7975	
ALT	168		21.54	18.05	4	140	18.79	24.29	153		27.21	19.37	5	124	24.11	30.3	0.007	
Cre	173		0.9	1.22	0.48	13.37	0.71	1.08	162		0.95	0.77	0.48	9.69	0.83	1.07	0.6413	
GFR	173		99.83	22.24	4.1	162.5	96.49	103.16	161		91.92	21.34	6.2	151.5	88.6	95.24	0.001	
UA	119		5.24	1.51	2.6	11.2	4.96	5.51	86		5.94	1.36	3.1	10.5	5.64	6.23	0.0008	

	control group eyes(N=540)								galucoma group eyes(N=588)								P-value	
OD/OS																	0.6784	Chisq
OD	273	50.6							290	49.3								
OS	267	49.4							298	50.7								
VA_MAR	540		0.11	0.18	-0.18	0.69	0.09	0.12	579		0.33	0.63	-0.41	3.91	0.28	0.38	<0.0001	
AL	540		25.18	1.71	21.59	32.53	25.04	25.33	587		25.7	2.19	20.18	33.55	25.52	25.88	<0.0001	
<24	148	27.4							144	24.6							<0.0001	Chisq
24-25.9	222	41.1							172	29.4								
≥26	170	31.5							270	46.1								
IOP	540		14.67	3.44	6	29	14.38	14.96	588		14.47	3.83	5	46	14.16	14.78	0.3622	
CCT	539		544.34	36.54	391	655	541.25	547.43	587		534.52	36.83	402	679	531.53	537.5	<0.0001	
VF: mean defect	112		-1.33	1.94	-11.4	1.45	-1.69	-0.97	570		-9.61	9.51	-33.28	31.88	-10.39	-8.82	<0.0001	
Macular Superior	528		50.26	4.78	28	59	49.85	50.67	556		42.76	7.59	24	58	42.13	43.39	<0.0001	
Macular Center	529		18.42	6.39	2	44	17.87	18.97	557		15.95	6.71	2	41	15.39	16.5	<0.0001	
Macular Inferior	529		49.69	4.98	22	60	49.26	50.12	548		40.3	8.18	17	57	39.62	40.99	<0.0001	
Disc Superior	535		51.79	4.87	28	63	51.38	52.2	559		38.3	11.06	12	62	37.38	39.22	<0.0001	
Disc Inferior	534		52.88	5.18	29	65	52.44	53.32	553		36.12	10.86	11	60	35.21	37.02	<0.0001	
RNFL																		
RNFL Superior	540		100.75	9.67	54	133	99.93	101.57	585		75.94	15.51	34	137	74.68	77.2	<0.0001	
RNFL Inferior	540		96.81	8.99	54	122	96.05	97.57	585		69.33	14.9	35	151	68.12	70.54	<0.0001	
GCC																		
GCC Superior	529		95.9	5.75	80	116	95.41	96.39	564		76.63	12.66	47	115	75.59	77.68	<0.0001	
GCC Inferior	529		95.32	5.73	73	114	94.83	95.81	564		70.66	12.65	48	131	69.62	71.71	<0.0001	
CD V.Ratio(%)	540		49.67	19.45	0	92	48.03	51.32	585		81.55	14.84	0	99	80.35	82.76	<0.0001	
Rim Area(0.01$mm^{3)}$	540		133.33	36.73	23	285	130.22	136.43	584		70.6	38.98	7	394	67.43	73.77	<0.0001	
Disc Area(0.01mm^2)	539		203.43	48.61	39	398	199.31	207.54	582		214.4	60.7	32	546	209.46	219.34	0.0008	
