# Supplementary material for: Vessel Density in the Macular and Peripapillary Areas in Preperimetric Glaucoma to Various Stages of Primary Open-Angle Glaucoma in Taiwan
Source: J Clin Med. 2021 Nov 23;10(23):5490. doi: 10.3390/jcm10235490 (PMC8658219; doi:10.3390/jcm10235490)
Supplement: Supplementary file 1 [file jcm-10-05490-s001.zip › Supplementary Table S2.pdf]

Demographics, Characteristics and Ocular Data of the Control and Glaucoma groups after Gender and Age Matching

	control group patients(N=200)									galucoma group patients(N=200)										
	N	(%)	Means	SD		min	max	95%CI		N	(%)		Means	SD	min	max	95%CI		P-value	
Age	200		47.39	15.17	47.39±15.17	20	81	45.27	49.51	200		49.27±14.67	49.27	14.67	20	80	47.22	51.32	0.0843	pair t-test
<40	62	31.0			62(31%)					51	25.5	51(25.5%)							0.2452	Mantel-Haenszel Chisq
40-60	101	50.5			101(50.5%)					107	53.5	107(53.5%)								
>=60	37	18.5			37(18.5%)					42	21.0	42(21%)								
Sex																			0.1864	Mantel-Haenszel Chisq
M	88	44.0			88(44%)					75	37.5	75(37.5%)								
F	112	56.0			112(56%)					125	62.5	125(62.5%)								
SBP	200		124.69	17.91	124.69±17.91	72	185	122.19	127.19	198		126.92±19.39	126.92	19.39	90	199	124.2	129.64	0.2341	independent t test
DBP	200		74.18	11.77	74.18±11.77	46	111	72.54	75.82	198		73.29±11.53	73.29	11.53	44	108	71.68	74.91	0.4481	independent t test
MAP	200		91.02	12.89	91.02±12.89	59.33	134	89.22	92.81	198		91.17±13.07	91.17	13.07	60.33	131	89.34	93	0.9072	independent t test
HR	200		78.44	13.12	78.44±13.12	49	121	76.6	80.27	198		76.31±12.41	76.31	12.41	45	117	74.57	78.05	0.0975	independent t test
TG	86		114.74	84.51	114.74±84.51	32	549	96.62	132.86	64		102.92±52.16	102.92	52.16	25	316	89.89	115.95	0.2932	independent t test
HDL	80		56.13	14.97	56.13±14.97	30	93	52.79	59.46	63		54.65±15.23	54.65	15.23	30	97	50.82	58.49	0.5628	independent t test
LDL	60		107.23	31	107.23±31	36	201	99.22	115.24	50		104.94±31.16	104.94	31.16	33	173	96.09	113.79	0.7007	independent t test
AC sugar	95		96.52	16.08	96.52±16.08	68	177	93.24	99.79	77		101.88±24.16	101.88	24.16	80	207	96.4	107.37	0.0969	independent t test
HbA1C	31		6.02	0.67	6.02±0.67	5	8.1	5.77	6.26	43		5.83±0.71	5.83	0.71	4.8	8.4	5.61	6.05	0.2499	independent t test
ALT	108		23.58	20.89	23.58±20.89	6	140	19.6	27.57	81		25.8±21.05	25.8	21.05	9	124	21.15	30.46	0.4722	independent t test
Cre	112		0.95	1.31	0.95±1.31	0.48	13.37	0.7	1.19	86		0.85±0.44	0.85	0.44	0.48	4.06	0.75	0.94	0.4488	independent t test
GFR	112		97.12	21.44	97.12±21.44	4.1	162.5	93.1	101.13	85		95.61±22.38	95.61	22.38	12.6	151.5	90.78	100.43	0.6313	independent t test
UA	69		5.53	1.77	5.53±1.77	2.8	11.2	5.11	5.96	48		5.56±1.48	5.56	1.48	3.1	10.5	5.13	5.99	0.9310	independent t test
	control group eyes(N=343)									galucoma group eyes(N=343)									P-value	
OD/OS																			0.7600	Chisq
OD	173	50.4			173(50.44%)					177	51.6	177(51.6%)								
OS	170	49.6			170(49.56%)					166	48.4	166(48.4%)								
VA_MAR	343		0.11	0.19	0.11±0.19	-0.18	0.69	0.09	0.13	340		0.28±0.57	0.28	0.57	-0.41	3.91	0.22	0.34	<0.0001	independent t test
AL	343		25.06	1.65	25.06±1.65	21.59	32.53	24.88	25.24	342		25.7±2.09	25.7	2.09	20.18	31.82	25.48	25.93	<0.0001	independent t test
<24	100	29.2			100(29.15%)					75	22.0	75(21.99%)							<0.0001	Chisq
24-25.9	145	42.3			145(42.27%)					101	29.6	101(29.62%)								
≥26	98	28.6			98(28.57%)					165	48.4	165(48.39%)								
IOP	343		14.86	3.64	14.86±3.64	6	29	14.47	15.24	343		14.66±4.14	14.66	4.14	5	46	14.22	15.1	0.5094	independent t test
CCT	343		547.06	33.17	547.06±33.17	419	655	543.54	550.58	342		535.35±37.86	535.35	37.86	410	679	531.32	539.37	<0.0001	independent t test
VF: mean defect	81		-1.24	1.82	-1.24±1.82	-6.49	1.45	-1.65	-0.84	332		-8.21±8.3	-8.21	8.3	-33.28	0.91	-9.1	-7.31	<0.0001	independent t test
Macular Superior	335		50.1	4.71	50.1±4.71	29	59	49.59	50.6	327		43.76±7.43	43.76	7.43	24	56	42.95	44.57	<0.0001	independent t test
Macular Center	335		18.73	6.35	18.73±6.35	2	34	18.05	19.41	328		16.36±7.04	16.36	7.04	2	41	15.59	17.12	<0.0001	independent t test
Macular Inferior	335		49.63	4.7	49.63±4.7	22	58	49.13	50.13	322		41.38±8.1	41.38	8.1	17	56	40.49	42.27	<0.0001	independent t test
Disc Superior	340		51.56	4.99	51.56±4.99	28	63	51.03	52.09	333		40.49±10.66	40.49	10.66	18	62	39.34	41.64	<0.0001	independent t test
Disc Inferior	339		52.68	5	52.68±5	30	65	52.15	53.22	330		37.88±10.48	37.88	10.48	11	60	36.75	39.02	<0.0001	independent t test
RNFL																				independent t test
RNFL Superior	343		99.95	9.63	99.95±9.63	54	133	98.93	100.98	343		78.17±15.52	78.17	15.52	39	137	76.52	79.82	<0.0001	independent t test
RNFL Inferior	343		96.05	9.06	96.05±9.06	54	122	95.09	97.01	343		71.95±15.07	71.95	15.07	37	151	70.35	73.55	<0.0001	independent t test
GCC																				independent t test
GCC Superior	335		95.58	5.54	95.58±5.54	80	116	94.98	96.17	332		78.44±12.47	78.44	12.47	47	115	77.09	79.79	<0.0001	independent t test
GCC Inferior	335		95.02	5.49	95.02±5.49	73	113	94.43	95.61	332		72.29±13.15	72.29	13.15	49	131	70.87	73.71	<0.0001	independent t test
CD V.Ratio(%)	343		51.22	18.62	51.22±18.62	0	92	49.24	53.2	343		79.19±16.01	79.19	16.01	0	99	77.49	80.89	<0.0001	independent t test
Rim Area(0.01mm$^{3)}$	343		131.1	36.93	131.1±36.93	34	285	127.17	135.02	342		75.91±39.82	75.91	39.82	7	394	71.67	80.14	<0.0001	independent t test
Disc Area(0.01mm^2)	342		203.51	49.35	203.51±49.35	104	398	198.27	208.76	341		212.05±61.54	212.05	61.54	32	546	205.49	218.6	0.0461	independent t test
