# Supplementary material for: Vessel Density in the Macular and Peripapillary Areas in Preperimetric Glaucoma to Various Stages of Primary Open-Angle Glaucoma in Taiwan
Source: J Clin Med. 2021 Nov 23;10(23):5490. doi: 10.3390/jcm10235490 (PMC8658219; doi:10.3390/jcm10235490)
Supplement: Supplementary file 1 [file jcm-10-05490-s001.zip › Supplementary Table S3.pdf]

Demographics, Clinical Characteristics and Ocular Data after Gender and Age Matching for Groups of Glaucomatous Subjects and Control Subjects
1:9 PS-matching for PPG groups and control groups

	control group eyes(N = 288)									preperimetric glaucoma group eyes(N = 32)										
	N	(%)	Means	SD		min	max	95%CI		N	(%)	Means	SD		min	max	95%CI		P-value	
Age	288		44.71	13.85	44.71±13.85	20	81	43.11	46.32	32		48.59	15.66	48.59±15.66	21	78	42.95	54.24	0.1230	pair ttest
<40	112	38.89			112(38.89%)					9	28.13			9(28.13%)					0.2358	Mantel-Haenszel Chi-squar
40-60	142	49.31			142(49.31%)					18	56.25			18(56.25%)						
>=60	34	11.81			34(11.81%)					5	15.63			5(15.63%)						
Sex																			1	Mantel-Haenszel Chi-squar
M	72	25			72(25%)					8	25			8(25%)						
F	216	75			216(75%)					24	75			24(75%)						
SBP	287		122.52	17.66	122.52±17.66	72	185	120.47	124.57	32		130.31	18.39	130.31±18.39	93	169	123.68	136.94	0.0282	
DBP	287		72.02	11.98	72.02±11.98	44	113	70.62	73.41	32		76.81	13.04	76.81±13.04	47	107	72.11	81.52	0.0341	
MAP	287		88.85	13.01	88.85±13.01	57.33	134	87.34	90.36	32		94.65	13.53	94.65±13.53	62.33	123	89.77	99.52	0.0179	
HR	287		79.41	12.41	79.41±12.41	49	121	77.97	80.85	32		75.47	9.82	75.47±9.82	60	97	71.93	79.01	0.0834	
TG	150		105.68	69.88	105.68±69.88	31	549	94.41	116.95	10		102.3	45.63	102.3±45.63	25	162	69.66	134.94	0.6449	Wilcoxon rank-sum test
HDL	146		55.91	13.5	55.91±13.5	30	93	53.7	58.12	10		55.2	12.4	55.2±12.4	39	85	46.33	64.07	0.7725	Wilcoxon rank-sum test
LDL	93		113.69	32.79	113.69±32.79	36	208	106.94	120.44	6		115.17	35.07	115.17±35.07	72	169	78.36	151.97	0.8895	Wilcoxon rank-sum test
AC sugar	159		94.11	14.26	94.11±14.26	68	177	91.87	96.34	14		93.43	8.05	93.43±8.05	80	108	88.78	98.08	0.7033	Wilcoxon rank-sum test
HbA1C	39		6.01	0.67	6.01±0.67	5	8.1	5.79	6.23	3		5.07	0.25	5.07±0.25	4.8	5.3	4.44	5.69	0.0157	Wilcoxon rank-sum test
ALT	174		20.24	17.05	20.24±17.05	4	140	17.69	22.79	14		32	21.91	32±21.91	9	82	19.35	44.65	0.0288	Wilcoxon rank-sum test
Cre	178		0.87	1.2	0.87±1.2	0.48	13.37	0.69	1.05	14		0.77	0.18	0.77±0.18	0.51	1.11	0.66	0.87	0.4944	Wilcoxon rank-sum test
GFR	178		100.93	21.34	100.93±21.34	4.1	162.5	97.77	104.08	14		99.19	16.55	99.19±16.55	78.4	136.2	89.63	108.74	0.3684	Wilcoxon rank-sum test
UA	123		5.1	1.34	5.1±1.34	2.6	9.8	4.87	5.34	7		5.29	1.26	5.29±1.26	3.2	6.5	4.12	6.45	0.5101	Wilcoxon rank-sum test
	control group eyes(N = 288)									preperimetric glaucoma group eyes(N = 32)									P-value	
OD/OS																			0.6814	Chi Squared test
OD	151	52.43			151(52.43%)					18	56.25			18(56.25%)						
OS	137	47.57			137(47.57%)					14	43.75			14(43.75%)						
VA_MAR	288		0.11	0.18	0.11±0.18	-0.18	0.69	0.09	0.13	32		0.21	0.64	0.21±0.64	-0.18	3.51	-0.02	0.44	0.3730	
AL	288		25.21	1.8	25.21±1.8	21.6	32.53	25	25.42	32		25.62	1.8	25.62±1.8	22.39	29.06	24.97	26.27	0.2241	
<24	78	27.08			78(27.08%)					7	21.88			7(21.88%)					0.0359	Chi Squared test
24-25.9	121	42.01			121(42.01%)					8	25			8(25%)						
⩾26	89	30.9			89(30.9%)					17	53.13			17(53.13%)						
IOP	288		14.63	3.37	14.63±3.37	6	29	14.24	15.02	32		14.91	3.31	14.91±3.31	9	22	13.71	16.1	0.6601	independent t-test
CCT	287		543.87	35.86	543.87±35.86	404	655	539.71	548.04	32		542.19	31.03	542.19±31.03	441	620	531	553.37	0.7984	independent t-test
VF: mean defect	63		-1.38	1.78	-1.38±1.78	-6.11	1.45	-1.82	-0.93	31		-1.37	1.53	-1.37±1.53	-5.02	0.91	-1.93	-0.81	0.9775	independent t-test
Macular Superior	283		50.14	4.81	50.14±4.81	29	58	49.57	50.7	32		47.28	5.11	47.28±5.11	34	54	45.44	49.12	0.0017	independent t-test
Macular Center	284		18.43	6.76	18.43±6.76	2	44	17.64	19.22	32		17.97	6.44	17.97±6.44	6	33	15.65	20.29	0.7158	independent t-test
Macular Inferior	284		49.46	5.13	49.46±5.13	22	60	48.87	50.06	31		46.13	4.83	46.13±4.83	35	55	44.36	47.9	0.0006	independent t-test
Disc Superior	287		51.71	5.16	51.71±5.16	28	63	51.11	52.31	32		47.09	6.37	47.09±6.37	33	60	44.8	49.39	<0.0001	independent t-test
Disc Inferior	285		52.73	5.41	52.73±5.41	29	65	52.1	53.36	31		48.48	5.2	48.48±5.2	35	58	46.58	50.39	<0.0001	independent t-test
RNFL																				
RNFL Superior	288		101.04	10.13	101.04±10.13	54	133	99.86	102.21	32		85.56	10.22	85.56±10.22	63	106	81.88	89.25	<0.0001	independent t-test
RNFL Inferior	288		96.94	9.39	96.94±9.39	54	122	95.85	98.03	32		85.09	9.8	85.09±9.8	68	105	81.56	88.63	<0.0001	independent t-test
GCC																				
GCC Superior	284		96	5.86	96±5.86	80	113	95.32	96.69	32		86.34	7.36	86.34±7.36	74	102	83.69	89	<0.0001	independent t-test
GCC Inferior	284		95.4	5.85	95.4±5.85	73	113	94.71	96.08	32		83.53	7.27	83.53±7.27	69	100	80.91	86.15	<0.0001	independent t-test
CD V.Ratio(%)	288		49.76	19.54	49.76±19.54	0	92	47.49	52.02	32		66.5	18.58	66.5±18.58	13	88	59.8	73.2	<0.0001	independent t-test
Rim Area(0.01mm$^{3)}$	288		133.34	37.2	133.34±37.2	23	285	129.03	137.66	32		109.25	63.74	109.25±63.74	45	394	86.27	132.23	0.0435	independent t-test
Disc Area(0.01mm^{2})	287		203.46	48.42	203.46±48.42	39	398	197.83	209.08	32		218.94	56.15	218.94±56.15	130	407	198.69	239.18	0.0925	independent t-test

Demographics, Clinical Characteristics and Ocular Data after Gender and Age Matching for Groups of Glaucomatous Subjects and Control

1:1 PS-matching for glaucoma eye (excluding PPG) and control groups

	control group eyes(N=314)									galucoma group eyes(N=314)										
	N	(%)	Means	SD		min	max	95%CI		N	(%)	Means	SD		min	max	95%CI		P-value	
Age	314		47.65	14.4	47.65±14.36	21	81	46.06	49.24	314		48.21	12.9	48.21±12.85	20	75	46.79	49.6	0.5203	
<40	81	25.8			81(25.8%)					95	30.3			95(30.25%)					0.9538	Mantel-Haenszel Chi Squared
40-60	177	56.37			177(56.37%)					150	47.8			150(47.77%)						
>=60	56	17.83			56(17.83%)					69	22			69(21.97%)						
Sex																			1	Mantel-Haenszel Chi Squared
M	156	49.68			156(49.68%)					156	49.7			156(49.68%)						
F	158	50.32			158(50.32%)					158	50.3			158(50.32%)						
SBP	314		126.2	18.4	126.21±18.36	72	185	124.17	128.25	312		125.94	17.5	125.94±17.5	90	182	123.99	128	0.8536	
DBP	314		75.41	12	75.41±12	44	111	74.08	76.75	312		74.53	11.7	74.53±11.73	44	110	73.22	75.8	0.3511	
MAP	314		92.35	13.2	92.35±13.24	57.33	130	90.87	93.82	312		91.67	12.5	91.67±12.51	60.33	124.33	90.27	93.1	0.5103	
HR	314		78.8	12.3	78.8±12.31	53	120	77.43	80.16	312		74.69	11.5	74.69±11.47	45	112	73.41	76	<0.0001	
TG	146		122.7	87.1	122.69±87.09	31	549	108.45	136.94	100		113.05	49.7	113.05±49.66	25	266	103.2	123	0.2718	
HDL	136		52.37	14.1	52.37±14.1	30	91	49.98	54.76	95		52.46	14.8	52.46±14.76	32	97	49.46	55.5	0.9604	
LDL	107		113.6	34.1	113.62±34.13	36	208	107.08	120.16	77		109.13	29.1	109.13±29.1	33	173	102.52	116	0.3512	
AC sugar	153		96.85	14.1	96.85±14.11	68	140	94.6	99.1	110		100.34	19.8	100.34±19.79	82	207	96.6	104	0.1155	
HbA1C	49		5.96	0.49	5.96±0.49	5	7.4	5.82	6.1	65		6.11	1.05	6.11±1.05	4.8	10.5	5.85	6.37	0.3312	
ALT	161		22.24	11.9	22.24±11.89	4	65	20.39	24.09	127		23.76	16.2	23.76±16.2	5	124	20.91	26.6	0.3785	
Cre	176		1.01	1.47	1.01±1.47	0.5	13.37	0.79	1.23	132		0.92	0.84	0.92±0.84	0.48	9.69	0.77	1.06	0.4715	
GFR	176		94.97	21.4	94.97±21.41	4.1	147.8	91.78	98.15	130		95.89	21.2	95.89±21.22	6.2	151.5	92.21	99.6	0.7089	
UA	116		5.7	1.62	5.7±1.62	3.1	9.8	5.4	6	66		5.87	1.34	5.87±1.34	3.1	9.1	5.54	6.2	0.4567	
	control group eyes(N=314)									galucoma group eyes(N=314)									P-value	
OD/OS																			0.5764	Chi Squared test
OD	160	50.96			160(50.96%)					153	48.7			153(48.73%)						
OS	154	49.04			154(49.04%)					161	51.3			161(51.27%)						
VA_MAR	314		0.11	0.18	0.11±0.18	-0.18	0.69	0.09	0.13	311		0.28	0.54	0.28±0.54	-0.41	3.91	0.22	0.34	<0.0001	
AL	314		25.12	1.78	25.12±1.78	21.59	32.53	24.93	25.32	313		25.68	2.09	25.68±2.09	20.18	31.86	25.45	25.9	0.0004	
<24	97	30.89			97(30.89%)					71	22.8			71(22.76%)					0.0016	Chi Squared test
24-25.9	118	37.58			118(37.58%)					100	32.1			100(32.05%)						
⩾26	99	31.53			99(31.53%)					141	45.2			141(45.19%)						
IOP	314		14.81	3.58	14.81±3.58	6	29	14.41	15.21	314		14.75	3.99	14.75±3.99	5	46	14.31	15.2	0.8383	
CCT	313		544	35.4	543.99±35.44	419	655	540.05	547.93	313		536.39	34.1	536.39±34.11	431	679	532.6	540	0.0065	
VF: mean defect	76		-1.24	1.87	-1.24±1.87	-6.49	1.45	-1.66	-0.81	306		-9.04	8.55	-9.04±8.55	-33.28	0.48	-10.01	-8.1	<0.0001	
Macular Superior	306		49.9	5.25	49.9±5.25	28	59	49.31	50.49	297		43.52	7.11	43.52±7.11	25	58	42.7	44.3	<0.0001	
Macular Center	306		18.61	6.34	18.61±6.34	2	34	17.89	19.32	298		16.13	6.96	16.13±6.96	2	41	15.33	16.9	<0.0001	
Macular Inferior	306		49.44	5.39	49.44±5.39	22	59	48.84	50.05	293		40.99	7.73	40.99±7.73	17	56	40.1	41.9	<0.0001	
Disc Superior	311		51.28	5.26	51.28±5.26	28	63	50.7	51.87	304		39.11	10.6	39.11±10.6	18	62	37.92	40.3	<0.0001	
Disc Inferior	310		52.4	5.43	52.4±5.43	30	64	51.8	53.01	303		36.57	10.2	36.57±10.19	14	60	35.42	37.7	<0.0001	
RNFL																				
RNFL Superior	314		100.8	9.99	100.75±9.99	54	133	99.64	101.85	314		76.03	16	76.03±16.04	34	137	74.25	77.8	<0.0001	
RNFL Inferior	314		96.26	8.93	96.26±8.93	54	120	95.27	97.25	314		69.33	15	69.33±15.03	36	151	67.67	71	<0.0001	
GCC																				
GCC Superior	306		95.79	5.42	95.79±5.42	82	116	95.18	96.4	302		76.63	12.2	76.63±12.19	47	105	75.25	78	<0.0001	
GCC Inferior	306		95.1	5.6	95.1±5.6	73	114	94.47	95.73	302		70.47	12.5	70.47±12.54	50	131	69.05	71.9	<0.0001	
CD V.Ratio(%)	314		51.52	19.4	51.52±19.36	0	92	49.37	53.67	314		80.73	15.6	80.73±15.64	0	99	78.99	82.5	<0.0001	
Rim Area(0.01mm$^{3)}$	314		130.8	37.1	130.83±37.05	34	285	126.72	134.95	313		72	39.1	72±39.09	7	282	67.65	76.4	<0.0001	
Disc Area(0.01mm^{2})	313		204.2	49.4	204.17±49.42	104	389	198.68	209.67	312		212.61	60.1	212.61±60.08	32	472	205.92	219	0.0557	

Demographics, Clinical Characteristics and Ocular Data after Gender and Age Matching for Groups of Glaucomatous Subjects and Control Subjects
1:5 PS-matching for glaucoma eye (excluding PPG) and PPG groups

	galucoma group eyes(N=310)									preperimetric glaucoma group eyes(N=62)										1/5
	N	(%)	Means	SD		min	max	95%CI		N	(%)	Means	SD		min	max	95%CI		P-value	
Age	310		50.57	14.1	50.57±14.05	20	80	49	52.1	62		46.87	12.2	46.87±12.24	21	78	43.76	49.98	0.3317	
<40	75	24.2			75(24.19%)					15	24.2			15(24.19%)					0.2032	Mantel-Haenszel Chi-square
40-60	163	52.6			163(52.58%)					40	64.52			40(64.52%)						
>=60	72	23.2			72(23.23%)					7	11.29			7(11.29%)						
Sex																			0.2315	Mantel-Haenszel Chi-square
M	190	61.3			190(61.29%)					43	69.35			43(69.35%)						
F	120	38.7			120(38.71%)					19	30.7			19(30.65%)						
SBP	308		128.95	18.6	128.95±18.62	90	205	126.87	131	62		129.5	16	129.53±15.98	93	169	125.47	133.59	0.8198	
DBP	308		75.97	12.3	75.97±12.25	44	110	74.6	77.3	62		78.6	12.5	78.6±12.45	47	113	75.43	81.76	0.1255	
MAP	308		93.63	13.1	93.63±13.08	60.33	140.33	92.17	95.1	62		95.58	12.7	95.58±12.65	62.33	131.3	92.36	98.79	0.2840	
HR	308		75.51	12.3	75.51±12.31	45	117	74.13	76.9	62		74.89	10.1	74.89±10.13	56	98	72.31	77.46	0.7075	
TG	94		116.86	57.2	116.86±57.22	39	316	105.14	129	23		108.6	45	108.61±44.97	25	239	89.16	128.06	0.5208	
HDL	90		50.41	15.5	50.41±15.49	30	103	47.17	53.7	24		52.04	11.7	52.04±11.69	38	85	47.11	56.98	0.6322	
LDL	73		113.42	31.7	113.42±31.68	33	193	106.03	121	19		119.2	27.4	119.16±27.36	72	173	105.97	132.34	0.4727	
AC sugar	110		104.19	22.5	104.19±22.54	80	200	99.93	108	27		95.37	8.07	95.37±8.07	80	112	92.18	98.56	0.0012	
HbA1C	71		6.05	0.88	6.05±0.88	4.8	10.5	5.84	6.26	14		5.4	0.57	5.4±0.57	4.5	6.6	5.07	5.73	0.0101	
ALT	123		25.71	18.3	25.71±18.28	5	124	22.44	29	27		31.11	21.7	31.11±21.73	9	96	22.52	39.71	0.1814	
Cre	130		0.94	0.81	0.94±0.81	0.48	9.69	0.8	1.08	28		0.85	0.18	0.85±0.18	0.51	1.23	0.78	0.92	0.268	
GFR	129		93.01	21.2	93.01±21.16	6.2	151.5	89.33	96.7	28		97.88	17.7	97.88±17.74	67.3	137.1	91	104.75	0.2595	
UA	72		5.97	1.36	5.97±1.36	3.1	10.5	5.65	6.29	17		5.9	1.31	5.9±1.31	3.2	8.1	5.23	6.57	0.8457	
	galucoma group eyes(N=310)									preperimetric glaucoma group eyes(N=62)									P-value	
OD/OS																			0.6764	Chi Squared test
OD	159	51.3			159(51.29%)					30	48.39			30(48.39%)						
OS	151	48.7			151(48.71%)					32	51.61			32(51.61%)						
VA_MAR	304		0.37	0.69	0.37±0.69	-0.18	3.91	0.29	0.44	62		0.25	0.6	0.25±0.6	-0.18	3.51	0.09	0.4	0.2026	
AL	310		25.6	2.39	25.6±2.39	20.18	33.55	25.34	25.9	62		25.85	1.89	25.85±1.89	22.39	29.3	25.37	26.33	0.3713	
<24	86	27.8			86(27.83%)					13	21.0			13(20.97%)					0.3722	Chi Squared test
24-25.9	87	28.2			87(28.16%)					16	25.81			16(25.81%)						
⩾26	136	44			136(44.01%)					33	53.23			33(53.23%)						
IOP	310		14.65	3.77	14.65±3.77	6	29	14.23	15.1	62		15.05	3.97	15.05±3.97	8	25	14.04	16.06	0.4488	
CCT	309		536.45	36	536.45±36.04	438	660	532.41	540	62		530.7	44.7	530.71±44.65	402	632	519.37	542.05	0.3443	
VF: mean defect	301		-10.34	9.03	-10.34±9.03	-33.23	1.44	-11.36	-9.3	58		-1.01	1.28	-1.01±1.28	-5.02	0.91	-1.34	-0.67	<0.0001	
Macular Superior	288		42.9	7.49	42.9±7.49	24	58	42.03	43.8	62		46.37	6.68	46.37±6.68	27	56	44.68	48.07	0.0008	
Macular Center	290		16.04	6.85	16.04±6.85	2	41	15.25	16.8	62		17.81	6.17	17.81±6.17	3	33	16.24	19.37	0.0620	
Macular Inferior	283		40.18	8	40.18±8	17	57	39.24	41.1	61		45.74	6.11	45.74±6.11	23	56	44.17	47.3	<0.0001	
Disc Superior	297		37.9	10.6	37.9±10.6	13	61	36.69	39.1	59		47.31	5.94	47.31±5.94	33	60	45.76	48.85	<0.0001	
Disc Inferior	293		35.4	10.3	35.4±10.32	14	60	34.21	36.6	59		47.41	5.09	47.41±5.09	35	58	46.08	48.73	<0.0001	
RNFL																				
RNFL Superior	307		75.06	15.4	75.06±15.38	34	137	73.33	76.8	62		86.61	10.3	86.61±10.25	63	110	84.01	89.22	<0.0001	
RNFL Inferior	307		67.8	13.4	67.8±13.44	36	118	66.29	69.3	62		81.5	10.7	81.5±10.7	55	105	78.78	84.22	<0.0001	
GCC																				
GCC Superior	295		76.26	13	76.26±12.95	47	115	74.77	77.7	62		85.94	7.96	85.94±7.96	69	102	83.91	87.96	<0.0001	
GCC Inferior	295		69.96	12.8	69.96±12.84	48	131	68.49	71.4	62		81.31	8.11	81.31±8.11	64	100	79.25	83.37	<0.0001	
CD V.Ratio(%)	307		82.53	13.4	82.53±13.39	8	99	81.02	84	62		70.52	14.8	70.52±14.76	13	88	66.77	74.26	<0.0001	
Rim Area(0.01mm$^{3)}$	307		69.81	37.9	69.81±37.89	15	282	65.56	74.1	62		98.08	50.8	98.08±50.76	44	394	85.19	110.97	<0.0001	
Disc Area(0.01mm^2)	306		216.4	59.5	216.4±59.45	37	546	209.71	223	62		212.8	48.8	212.82±48.78	130	407	200.44	225.21	0.6572	
