# Supplementary material for: Vessel Density in the Macular and Peripapillary Areas in Preperimetric Glaucoma to Various Stages of Primary Open-Angle Glaucoma in Taiwan
Source: J Clin Med. 2021 Nov 23;10(23):5490. doi: 10.3390/jcm10235490 (PMC8658219; doi:10.3390/jcm10235490)
Supplement: Supplementary file 1 [file jcm-10-05490-s001.zip › Supplementary Table S4.pdf]

One Way Analysis of OCTA parameters and OCT measurement among the glaucoma severity groups

	control group(0) (N=540)							PPG (1,2,1+2) group(1) (N=67)							VF> -6dB(2) (N=224)							-12dB<VF≤ -6dB(3) (N=103)							VF≤ -12dB(4) (N=194)							ANOVA	
	N	Mean/N	SD/%	min	max	95%CI		N	Mean/N	SD/%	min	max	95%CI		N	Mean/N	SD/%	min	max	95%CI		N	Mean	SD	min	max	95%CI		N	Mean	SD	min	max	95%CI		P-value	Tukey test
Macular Superior	528	50.3	4.8	28.0	59.0	49.9	50.7	67	46.6	6.5	27	56	45	48	216	46.1	6.0	29	58	45	47	97	41.2	6.6	25	56	40	43	176	38.1	7.5	24	58	37	39	<0.0001	0>1,2>3>4
Macular Center	529	18.4	6.4	2.0	44.0	17.9	19.0	67	18.1	6.1	3	33	17	20	216	17.0	6.6	2	37	16	18	97	14.7	6.1	3	41	13	16	177	14.5	7.0	2	38	14	16	<0.0001	0>2>3,4; 1>3,4
Macular Inferior	529	49.7	5.0	22.0	60.0	49.3	50.1	66	46.0	5.9	23	56	45	47	215	43.7	6.8	26	55	43	45	96	38.0	7.2	25	56	37	39	171	35.2	7.7	17	57	34	36	<0.0001	0>1,2>3>4
Disc Superior	535	51.8	4.9	28.0	63.0	51.4	52.2	64	47.4	5.8	33	60	46	49	218	43.6	8.4	14	61	42	45	93	37.1	10.2	12	62	35	39	184	29.5	9.2	13	54	28	31	<0.0001	0>1>2>3>4
Disc Inferior	534	52.9	5.2	29.0	65.0	52.4	53.3	64	47.6	5.0	35	58	46	49	220	41.5	8.1	18	60	40	43	89	33.2	9.2	11	56	31	35	180	26.9	7.8	14	54	26	28	<0.0001	0>1>2>3>4
RNFL																																					
RNFL Superior	540	100.8	9.7	54.0	133.0	99.9	101.6	67	86.8	10.4	63	110	84	89	223	81.2	13.5	40	137	79	83	103	74.0	14.6	47	120	71	77	192	67.1	14.9	34	108	65	69	<0.0001	0>1>2>3>4
RNFL Inferior	540	96.8	9.0	54.0	122.0	96.1	97.6	67	82.0	10.7	55	105	79	85	223	74.6	12.8	48	151	73	76	103	65.2	14.0	37	121	62	68	192	61.1	13.4	35	111	59	63	<0.0001	0>1>2>3>4
GCC																																					
GCC Superior	529	95.9	5.8	80.0	116.0	95.4	96.4	67	86.0	7.8	69	102	84	88	216	81.1	10.2	57	106	80	82	98	73.9	12.6	53	101	71	76	183	69.4	12.4	47	115	68	71	<0.0001	0>1>2>3>4
GCC Inferior	529	95.3	5.7	73.0	114.0	94.8	95.8	67	81.5	8.0	64	100	80	83	216	75.0	12.1	54	131	73	77	98	65.9	10.4	48	99	64	68	183	64.2	11.1	50	119	63	66	<0.0001	0>1>2>3>4
CD V.Ratio(%)	540	49.7	19.5	0.0	92.0	48.0	51.3	67	69.5	16.0	13	88	66	73	223	77.5	14.0	0	98	76	79	103	83.4	13.7	0	98	81	86	192	89.5	11.0	8	99	88	91	<0.0001	0<1<2<3<4
Rim Area(0.01mm$^{3)}$	540	133.3	36.7	23.0	285.0	130.2	136.4	67	100.1	50.2	44	394	88	112	222	78.5	33.9	23	260	74	83	103	69.8	38.2	7	282	62	77	192	51.6	30.3	8	175	47	56	<0.0001	0>1>2,3>4
Disc Area(0.01mm^2)	539	203.4	48.6	39.0	398.0	199.3	207.5	67	213.9	49.8	130	407	202	226	221	209.5	51.8	99	453	203	216	102	221.0	81.9	32	546	205	237	192	216.7	60.6	37	419	208	225	0.01	3,4>0
Age	540	44.4	13.8	20.0	81.0	43.2	45.6	67	45.7	12.7	21	78	43	49	224	48.3	12.2	20	79	47	50	103	51.3	12.8	26	78	49	54	194	56.1	14.5	22	80	54	58	<0.0001	0<2,3；4>0,1,2,3
AL	540	25.2	1.7	21.6	32.5	25.0	25.3	67	25.9	1.8	22	29	25	26	224	25.9	1.8	21	32	26	26	103	26.3	2.8	21	34	26	27	193	25.1	2.2	20	31	25	25	<0.0001	1,2,3>0；1,2,3>4
Sex																																				0.46	
M		169	31.30						43	64.18						137	61.16						69	67.7						132	68.0						
F		371	68.70						24	35.82						87	38.84						33	32.4						62	32.0						
