# Supplementary material for: Vessel Density in the Macular and Peripapillary Areas in Preperimetric Glaucoma to Various Stages of Primary Open-Angle Glaucoma in Taiwan
Source: J Clin Med. 2021 Nov 23;10(23):5490. doi: 10.3390/jcm10235490 (PMC8658219; doi:10.3390/jcm10235490)
Supplement: Supplementary file 1 [file jcm-10-05490-s001.zip › Supplementary Table S5.pdf]

Comparison of advanced or terminal glaucoma with OCTA parameters and OCT measurement

(N = eye number)	Late-stage Glaucoma, VF≤-12dB (N=194)					Advanced Glaucoma, -12dB≤VF< -30 (N=158)					Terminal Glaucoma , -VF≤-30dB (N=36)						
	N	Mean	SD	min	max	N	Mean	SD	min	max	N	Mean	SD	min	max	P-value	indepented t test
Macular Superior	176	38.08	7.51	24.00	58.00	144	38.13	7.44	24.00	58.00	32	37.84	7.93	25.00	53.00	0.8449	
Macular Center	177	14.54	7.04	2.00	38.00	146	13.98	6.48	2.00	35.00	31	17.19	8.88	4.00	38.00	0.0638	
Macular Inferior	171	35.23	7.67	17.00	57.00	143	35.15	7.56	17.00	57.00	28	35.64	8.36	19.00	53.00	0.7587	
Disc Superior	184	29.50	9.24	13.00	54.00	151	29.68	8.84	13.00	52.00	33	28.70	11.02	18.00	54.00	0.6353	
Disc Inferior	180	26.92	7.82	14.00	54.00	148	26.86	7.33	14.00	54.00	32	27.16	9.92	14.00	47.00	0.8760	
RNFL																	
RNFL Superior	192	67.14	14.85	34.00	108.00	156	66.99	13.61	34.00	106.00	36	67.75	19.56	39.00	108.00	0.8269	
RNFL Inferior	192	61.09	13.36	35.00	111.00	156	60.94	13.20	35.00	111.00	36	61.72	14.21	37.00	86.00	0.7532	
GCC																	
GCC Superior	183	69.39	12.42	47.00	115.00	149	69.58	11.95	50.00	115.00	34	68.56	14.44	47.00	100.00	0.6673	
GCC Inferior	183	64.20	11.05	50.00	119.00	149	63.96	10.89	51.00	119.00	34	65.26	11.81	50.00	103.00	0.5358	
CD V.Ratio(%)	192	89.49	11.01	8.00	99.00	156	90.04	9.79	8.00	99.00	36	87.11	15.17	29.00	99.00	0.2740	
Rim Area(0.01mm$^{3)}$	192	51.63	30.30	8.00	175.00	156	49.92	25.06	8.00	175.00	36	59.03	46.49	15.00	172.00	0.2621	
Disc Area(0.01mm^2)	192	216.66	60.55	37.00	419.00	156	217.80	59.97	37.00	419.00	36	211.69	63.65	102.00	395.00	0.5868	
AL	193	25.05	2.19	20.18	31.46	158	25.07	2.19	20.18	31.46	35	24.98	2.24	21.33	30.47	0.8176	
VF	181	-21.69	6.40	-33.28	-12.02	158	-20.24	5.49	-29.68	-12.02	23	-31.71	0.87	-33.28	-30.01	<0.0001	
VA_MAR	185	0.60	0.87	0.00	3.91	158	0.55	0.79	0.00	3.91	27	0.91	1.20	0.00	3.69	0.1478	
Age	194	56.14	14.49	22.00	80.00	158	56.06	14.44	22.00	80.00	36	56.47	14.91	23.00	80.00	0.8790	
Male,%	132(68.04)					110(69.62)					22(61.11)					0.3231	
